# Antibiotic Treatment and Age Are Associated With *Staphylococcus aureus* Carriage Profiles During Persistence in the Airways of Cystic Fibrosis Patients

**DOI:** 10.3389/fmicb.2020.00230

**Published:** 2020-02-26

**Authors:** Corinna Westphal, Dennis Görlich, Stefanie Kampmeier, Susann Herzog, Nadja Braun, Carina Hitschke, Alexander Mellmann, Georg Peters, Barbara C. Kahl, Staphylococcal CF Study Group, Sibylle Junge.

**Affiliations:** Department of Paediatric Pulmonology and Neonatology, Medizinische Hochschule Hannover, Hannover, Germany; Clinical Research Group, Department of Paediatric Pulmonology and Neonatology, Medizinische Hochschule Hannover, Hannover, Germany; Burkhard Tümmler. CF-Center Innsbruck, Department of Paediatrics, University Hospital, Austria; Department of Paediatrics, University Hospital Münster, Münster, Germany; Department of Paediatrics, University Hospital Münster, Münster, Germany; Ruhr University Paediatric Clinic at St Josef Hospital, Bochum, Germany; Department of Paediatrics, University Hospital Essen, Essen, Germany; Paediatricians “Kinderärztliche Ambulanz” Hamburg, Germany; CF Center, Department of Paediatrics, University Clinics Jena, Jena, Germany; Pediatric Pulmonology/Cystic Fibrosis, Brandenburg Medical School (MHB) University. Brandenburg an der Havel, Germany; Department of Paediatrics, University Clinics Dresden, Dresden, Germany; Department of Pediatrics, University Clinics Tübingen, Tübingen, Germany; Department of Paediatric Pulmonology and Immunology, Charité – Universitätsmedizin Berlin, Campus Virchow Klinikum, Berlin, Germany; University Hospital Halle, Halle, Germany: Bettina Wollschläger. Children’s Hospital Osnabrück, Osnabrück, Germany; Department of Paediatrics, University of Düsseldorf, Düsseldorf, Germany; Park Schönefeld Clinics Kassel, Germany; Ruhrlandklinik Essen, Germany; University Clinics Leipzig, Germany; ^1^Institute of Medical Microbiology, University Hospital Münster, Münster, Germany; ^2^Institute of Biostatistics and Clinical Research, University Hospital Münster, Münster, Germany; ^3^Institute of Hygiene, University Hospital Münster, Münster, Germany

**Keywords:** *Staphylococcus aureus*, cystic fibrosis, persistent infection, *spa-*typing, clonal lineages, airway infection

## Abstract

**Background:**

*Staphylococcus aureus* is one of the most isolated pathogens from the airways of cystic fibrosis (CF) patients. There is a lack of information about the clonal nature of *S. aureus* cultured from CF patients and their impact on disease. We hypothesized that patients would differ in their clinical status depending on *S. aureus* clonal carriage profiles during persistence.

**Methods:**

During a 21-months prospective observational multicenter study (Junge et al., 2016), 3893 *S. aureus* isolates (nose, oropharynx, and sputa) were cultured from 183 CF patients (16 German centers, 1 Austrian center) and subjected to *spa*-sequence typing to assess clonality. Data were associated to lung function, age, gender, and antibiotic treatment by multivariate regression analysis.

**Results:**

Two hundred and sixty-five different *spa*-types were determined with eight prevalent *spa*-types (isolated from more than 10 patients): t084, t091, t008, t015, t002 t012, t364, and t056. We observed different carriage profiles of *spa*-types during the study period: patients being positive with a prevalent *spa*-type, only one, a dominant or related *spa*-type/s. Patients with more antibiotic cycles were more likely to be positive for only one *spa*-type (*p* = 0.005), while older patients were more likely to have related (*p* = 0.006), or dominant *spa*-types (*p* = 0.026). Two percent of isolates were identified as methicillin-resistant *S. aureus* (MRSA) and evidence of transmission of clones within centers was low.

**Conclusion:**

There was a significant association of antibiotic therapy and age on *S. aureus* carriage profiles in CF patients indicating that antibiotic therapy prevents acquisition of new clones, while during aging of patients with persisting *S. aureus*, dominant clones were selected and mutations in the *spa-*repeat region accumulated.

## Introduction

Cystic fibrosis (CF) is a life limiting genetic disease, which especially affects the lungs of CF patients with mucus retention and chronic bacterial infection of the airways leading to decreased lung function and reduced life expectancy (Elborn, 2016). *Staphylococcus aureus* is one of the earliest pathogens, which can be isolated from the airways of CF infants already (Cystic Fibrosis Foundation Patient Registry, 2018; European Cystic Fibrosis Society Patient Registry, 2018). *S. aureus* has a clonal population structure (Lindsay et al., 2006), is equipped with many virulence factors and can persist in the airways of CF patients for extended periods (Kahl et al., 2003; Schwerdt et al., 2018).

Several studies showed that in young CF patients, there is an increased lower airway inflammation with neutrophilic inflammation and pro-inflammatory cytokines and more clinical disease in case of *S. aureus* cultures compared to *S. aureus*-negative patients (Sagel et al., 2009; Gangell et al., 2011; Wong et al., 2013). However, there is less knowledge about the impact of *S. aureus* in older patients. In a prospective longitudinal multicenter study including 195 patients with persistent *S. aureus* cultures, we recently showed that in CF patients, who were older than 6 years, independent risk factors for worse lung function were high bacterial density in oropharyngeal cultures, exacerbations, elevated IL-6 levels, the presence of *S. aureus* small colony variants (SCVs), and co-infection with *Stenotrophomonas maltophilia* (Junge et al., 2016).

To determine the clonality of *S. aureus*, sequencing of the variable number of tandem repeat (VNTR) region of protein A, (SpA) *spa*-typing, represents an elegant, easy to perform, and low cost method compared to the more sophisticated and more cost intensive whole genome sequencing (WGS) (Harmsen et al., 2003; Koreen et al., 2004; O’Hara et al., 2016). *Spa-*types are assigned according to the sequence of base pairs within the repeats, which mostly consist of 24 base pairs, and the numbers of repeats, which range from 1 to 27 numbers as presented on the SpaServer^1^. It is also possible to cluster *spa-*types into related clonal complexes (*spa* CC) with defined common ancestors depending on their repeat composition (Mellmann et al., 2007). Interestingly, during persistence of *S. aureus* within CF airways, it has been shown that mutations occur in this region with deletions and duplications of repeats or point-mutations within repeats, leading to different *spa-*types (Kahl et al., 2005), which are closely related according to *spa* CCs. The relatedness of such clones with different *spa-*types, but very similar repeat successions was verified by pulsed-field gel electrophoresis (Kahl et al., 2005), multi-locus sequence typing (MLST) (Hirschhausen et al., 2013) or WGS (Schwartbeck et al., 2016). In different studies, we now confirmed that persistent *S. aureus* isolates persisting in the airways of CF patients, which are assigned to the same or related *spa*-types, which differed in their VNTR region by various mutations as outlined above, were confirmed to belong to the same clone by WGS (Schwartbeck et al., 2016; Langhanki et al., 2018; Herzog et al., 2019). Therefore, it seems that *spa*-sequence typing is a suitable method to analyze the relatedness of *S. aureus* isolates. Also, the VNTR region has been shown to be implicated in the regulation of inflammation (Martin et al., 2009) by its ability to modulate the pro-inflammatory response of SpA (Gómez et al., 2004) depending on the number of repeats (Garofalo et al., 2012).

There is a lack of knowledge about the *S. aureus* clones, which reside in the airways of CF patients and their dynamics during persistence. In this study, we determined the clonality of *S. aureus* isolates (*n* = 3893), which were cultured during a prospective long-term observational multicenter study (Junge et al., 2016), by *spa*-typing.

We hypothesized that lung disease of patients would differ depending on the carriage profiles of *S. aureus* depending on the clonality of isolates within the airways during our prospective study.

## Materials and Methods

### Patients, Specimens, and Bacteria

*Staphylococcus aureus* isolates (*n* = 3963), which were collected during a prospective 21-months multicenter study from 195 CF patients from 16 CF centers in Germany and 1 center in Austria (Junge et al., 2016), were used. Inclusion criteria were persistent *S. aureus* cultures a year before recruitment and being older than 6 years to be able to perform lung function tests. Exclusion criteria were chronic *Pseudomonas aeruginosa* or *Burkholderia cepacia* airway cultures. Specimens from nose, throat, and sputum were sent to the central study laboratory in Münster, where microbiological cultures were performed according to the requirements for CF airway cultures (Hogardt et al., 2006). *S. aureus* isolates were distinguished regarding size (normal/SCV phenotype), hemolysis (no hemolysis/weak/strong), and pigmentation (gray/white/yellow) on Columbia blood agar (Becton Dickinson, Heidelberg, Germany) incubated at 37°C, and on Schaedler agar (Becton Dickinson, Heidelberg, Germany) incubated at 37°C at 5% CO_2_. All *S. aureus* isolates with different phenotypes including hemolytic, non-hemolytic isolates, different pigmented isolates, and different size of isolates (SCVs, normal) were stored at −80°C and subjected to *spa*-sequence typing. For this study, all isolates were included in the further analysis.

### Susceptibility Testing

All *S. aureus* isolates were subjected to susceptibility testing. Normal isolates were tested by VITEK 2 system (bioMérieux), and SCVs by agar diffusion testing on Columbia blood agar due to the requirements of SCVs for thymidine (Hogardt et al., 2006).

### Antibiotic Treatment

In case report forms (CRFs), physicians reported antibiotic treatment of patients. For this analysis, only antibiotics directed against *S. aureus* were evaluated: first and second generation cephalosporins, antistaphylococcal penicillins, aminoglycosides, sulfamethoxazole/trimethoprim, clindamycin, rifampin.

### *Spa*-Typing

*Spa*-sequence typing was performed by amplification of the variable region of protein A by PCR with ensuing sequencing according to Harmsen et al. (2003). *Spa-*types were assigned according to the Ridom StaphType software (Ridom GmbH, Würzburg, Germany).

### BURP

By using the Based Upon Repeat Pattern method (BURP, Ridom StaphType software, Ridom GmbH, Würzburg, Germany) (Mellmann et al., 2007), we examined the clonal relatedness of *spa-*types for each individual patient as well as for the entire collection of isolates within and between each center.

### Whole Genome Sequence-Based Typing

To uncover the genetic relationships of the *S. aureus* isolates, a subset of strains (Supplementary Table S5) was compared via WGS-based typing using the Illumina MiSeq platform (Illumina Inc., San Diego, CA, United States) (Mellmann et al., 2016). After quality trimming, coding core genome regions were compared in a gene-by-gene approach (core genome multilocus sequence typing, cgMLST) using the SeqSphere+ software version 6.0.0 (Ridom GmbH, Münster, Germany) and the published *S. aureus* cgMLST target scheme (Leopold et al., 2014). To display the clonal relationship of genotypes, the minimum spanning tree algorithm was applied using the same software. Genotypes differing in ≤24 alleles were rated as closely related. For backwards compatibility with classical molecular typing the *spa*-types were extracted from the WGS data *in silico*.

### Statistical Analysis

We used SPSS (v.25, IBM) and SAS for the statistical tests and set the local significance level at α < 0.05. We used Mann–Whitney *U-*tests and logistic regression to run the tests. All models were adjusted to age and gender.

In addition to *spa-*types, we analyzed the categories age, gender, percentage of visits with antistaphylococcal antibiotics, percentage of visits with exacerbation, and the mean lung function measured as forced expiratory volume in 1 s in percent (FEV_1_%) predicted. We computed the variables as follows: The percentage of visits with antibiotics in relation to all visits of each patient (AB_percentage) and the percentage of visits with exacerbations in relation to all visits of each patient. We computed the mean lung function (mean FEV_1_% predicted) according to Quanjer et al. (2012). For the distribution of patients into the different *S. aureus* profiles, *spa-*types of all *S. aureus* isolates collected at all visits of individual patients were analyzed together.

## Results

One hundred and eighty-three of one hundred ninety-five recruited CF patients remained *S. aureus* positive throughout the study with at least 50% of cultures being culture positive for *S. aureus* indicating persistent infection. In 1120 of 1278 visits (range 1–18, mean seven visits per patient), *S. aureus* was cultured (88%) from the airway specimens. From 1929 samples, 3893 different *S. aureus* isolates were cultured with a mean number of isolates of 21 per patient (range of 1–83), Supplementary Table S1. There was a difference in the number of *spa-*types dependent on the site (*p* < 0.001) with the fewest number of different *spa-*types in sputa, followed by nose and throat (Supplementary Table S1).

### Population Structure of *S. aureus* Isolates as Assessed by *spa*-Typing

The 3893 *S. aureus* isolates could be assigned to 265 different *spa-*types. In each patient an average of 3.21 *spa-*types (range 1–12) was observed. For 7 of the 3893 isolates, no *spa-*type could be determined. These isolates were defined as non-typable.

**FIGURE 1:**
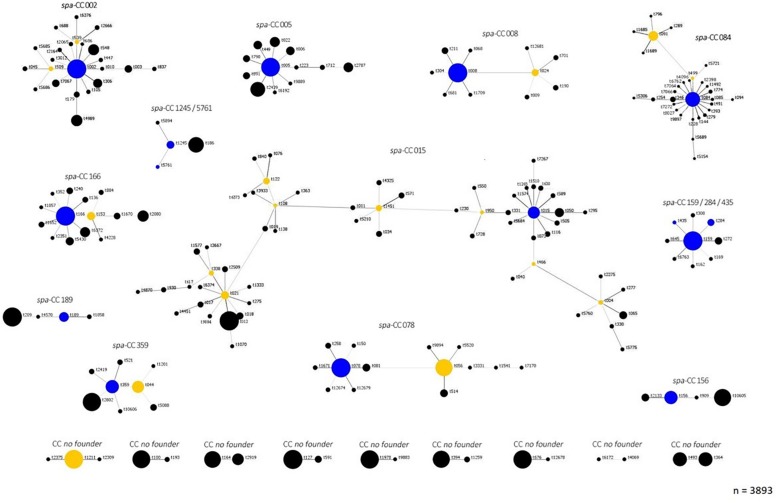
Population structure according to *spa* clonal complexes (CC) of *S. aureus* CF isolates. This figure demonstrates the clonal relatedness of all 265 *spa*-types of the 3893 *S. aureus* study across the 17 participating centers consisting of 16 German and 1 Austrian center. The analysis of the relationship of *spa*-types was performed based on the BURP algorithm as implemented in the Ridom StaphType software. 192 of the *spa*-types could be assigned to 12 CC: CC015, CC02, CC05, CC08, CC084, CC166, CC189, CC1245/5761, CC159/284/435, CC359, CC078, and CC156. The main founder *spa*-type is shown in blue in the middle of each CC, the sub founder marked in yellow, which leads to the following branch. All descendant *spa*-types are grouped around their respective founder. The size of each circle demonstrates the number of *S. aureus* isolates with the respective *spa*-type. For 19 *spa*-types, the BURP algorithm could determine no founder *spa*-type and therefore no clonal complex. These *spa*-types are presented as “no founder” and appear at the bottom of the figure. Thirty-six *spa*-types could not be related to other *spa*-types in our study. Therefore, BURP assigned them as singletons (dots not shown in this figure). *Spa*-types with four or less repeats were excluded from the analysis (*n* = 18, <1% of all *spa*-types), since no reliable information about the phylogenetic relatedness can be inferred. Singletons: t099, t185, t246, t370, t377, t488, t647, t746, t803, t1345, t1406, t1416, t1491, t1707, t2441, t2553, t2845, t3258, t5152, t5682, t5683, t5688, t5690, t5758, t5759, t6191, t6193, t6194, t6195, t6375, t7271, t9886, t9887, t9888, t9896, t12680. Excluded *spa*-types: t026, t103, t129, t227, t362, t390, t524, t559, t605, t693, t779, t1050, t1544, t1991, t2383, t3745, t5687, t7065.

To visualize the population structure of the study *S. aureus* isolates, all isolates were grouped into clonal complexes by BURP analysis, which compares the base sequence of the repeat region of the individual *spa*-types. The population structure of all isolates revealed a highly diverse, but also clonal population structure of *S. aureus* with 192 of 265 *spa*-types (72%) belonging to 12 *spa* CCs with related repeat regions, while 36 were specified as singletons without relation to any other *spa-*type in this study (Figure 1). There were eight prevalent *spa*-types, which were isolated from more than 10 patients: t084, t091, t008, t015, t002, t012, t346, and t056, Table 1. Further information of the number of patients, *S. aureus* isolates, *spa-*types and *spa* CCs of the individual centers is given in Supplementary Table S2 and Supplementary Figures S1–S17.

**TABLE 1:** Patients with prevalent *spa-*types.

*Spa-type*	*Spa* clonal complex	Isolates (*n*)	Percentage of all isolates (%)	Patients (*n*)	Percentage of patients^1^ (%)	Patients with persistence^2^ (*n*)	Percentage of patients with persistence (%)	Centers^3^ (*n*)
							
t084	CC084	310	7.8	36	19	18	50	16
t091	CC084	178	4.5	27	14	11	40	14
t008	CC008	138	3.5	18	9	11	61	10
t015	CC015	112	2.8	18	9	7	39	12
t002	CC002	108	2.8	16	9	7	44	9
t012	CC015	208	5.2	15	8	9	60	8
t346	CC084	72	1.8	14	8	3	21	9
t056	CC078	75	1.9	12	7	5	42	9

*^1^Percentage of all patients with isolation of that prevalent *spa*-type. ^2^At least 50% of specimen of patients being positive for that prevalent *spa*-type. ^3^Number of centers, in which the prevalent *spa*-type was isolated.*

### Patients Distinguished According to Special Carriage Profiles of *spa*-Types

Our prospective longitudinal study allowed observing different dynamics of *spa*-types within patients as a *post hoc* analysis of our data. Therefore, we classified patients according to the different carriage profiles of their *spa*-types for the subsequent analysis. According to our definitions, the classification of patients to the different carriage profiles is not necessarily exclusive, Supplementary Table S3. We created models with logical influence structures and analyzed the relation between the different carriage profiles and the categories age, gender, percentage of visits with antibiotics, percentage of visits with exacerbation, and FEV_1_% predicted, Supplementary Table S3.

#### Carriage Profile 1 – Prevalent *spa*-Types

We defined a *spa*-type as prevalent, if the *spa-*type was isolated from more than 10 patients. Eight *spa*-types were more prevalent than all other *spa*-types: t084, t091, t008, t015, t002, t012, t346, and t056 (Table 1). To be assigned to this group, at least in 50% of visits, prevalent *spa*-types had to be present in patients’ specimens (*n* = 68). This group was compared to patients without persisting prevalent *spa*-types (*n* = 115).

#### Carriage Profile 2 – Patients With Only One *spa-*Type

In some patients, only *S. aureus* isolates belonging to one *spa*-type were cultured during the entire study. Another single isolate with a different *spa*-type in one respiratory specimen at one visit was accepted. There were 64 patients with only one *spa*-type compared to patients with several *spa*-types (*n* = 119).

#### Carriage Profile 3 – Dominant *spa*-Types

A dominant *spa*-type was defined as a *spa*-type, which was traceable throughout all visits of the patient with more than 50% of all isolates of this individual patient (*n* = 65). Other clones could occur but were not observed persistently throughout the study period. Patients with dominant *spa*-types were compared to patients without dominant *spa*-types (*n* = 54).

#### Carriage Profile 4 – Related *spa*-Types

By BURP analysis, all *S. aureus* isolates of patients were grouped according to the repeat sequence of their *spa-*types and their relatedness. To be assigned to the group of patients with related *spa*-types, in at least 50% of visits of these patients, isolates with related *spa*-types had to be present. The group of patients with related *spa*-types (*n* = 33, exemplified for six patients in Table 2, for all patients in Supplementary Table S4) was compared to patients without related *spa*-types (*n* = 86). Mutations observed in isolates with related *spa*-types were: deletions (*n* = 24), duplications (*n* = 14), point-mutations (*n* = 12), and combined mutations (*n* = 6, Table 2 and Supplementary Table S4).

**TABLE 2:** Mutations within the VNTR region of related *spa*-types.

Patient	All clones^1^	Related clones^2^	Non-related^3^	Percentage of related clones^4^ (%)	Number of isolates^5^	*Spa*-type^6^	VNTR region^7^	Mutations^8^	Repeat^9^	Nucleotide sequence of the repeat region^10^
C1P4	5	4	1	80	20	**t050**	08-16-02-16-34-34-17-34-**16-**34			
					1	t295	08-16-02-16-34-34-17-34-34	del		
					2	**t008**	11-**19**-12-21-17-34-24-34-22-25			
					1	t024	11-12-21-17-34-24-34-22-25	del		
C1P7	9	5	4	56	1	**t277**	09-**20**-16-**13**-13-17-34-16-34		r20	AAAGAAGACAACAA**C**AAACCTGGC
					1	t040	09-**02**-16-13-17-34-16-34	del and pm	r02	AAAGAAGACAACAA**A**AAACCTGGC
					13	t004	09-02-16-13-13-17-34-16-34	pm		
					7	**t346**	07-23-12-34-12-**12**-23-02-12-23		r12	AAAGAAGACA**A**CAACAAGCCTGGT
					1	t2398	07-23-12-34-12-**66**-23-02-12-23	pm	r66	AAAGAAGACA**G**CAACAAGCCTGGT
C2P6	3	2	1	67	4	**t084**	07-23-12-34-34-12-12-23-02-12-23		r12	AAAGAAGACAACAACAAGCCTGG**T**
					1	t4096	07-23-**21**-12-34-34-12-12-23-02-12-23	dupl and pm	r21	AAAGAAGACAACAACAAGCCTGG**C**
C2P9	7	3	4	43	26	**t084**	07-23-12-34-**34**-12-**12**-23-02-12-23			
					1	t346	07-23-12-34-12-12-23-02-12-23	del		
					1	t085	07-23-12-34-34-12-23-02-12-23	del		
C3P3	4	2	2	50	7	**t078**	04-21-12-41-20-17-12-**12**-17			
					6	t081	04-21-12-41-20-17-12-17	del		
C3P9	5	2	3	40	13	**t499**	07-23-12-12-34-12-12-23-02-12-23			
					1	t9897	07-23-12-12-34-12-12-**12**-23-02-12-23	dupl		

*^1^All clones: all different *spa-*types isolated from the airways of this patient. ^2^Related clones: number of *spa*-types, which evolved most likely due to mutational events in the “variable number of repeat” region of spa during persistence. ^3^Non-related clones: number of additional clones with *spa*-types characterized by a non-related repeat region of *spa.*^4^Percentage of related clones: percentage of isolates with related *spa*-types.^5^Number of isolates with the respective *spa*-types. ^6^*Spa*-type: the different *spa*-types of patients with related *spa*-types; ancestor strains are marked in bold. ^7^VNTR-region: the sequence of the repeats within the VNTR-region; the mutated repeats are marked in bold in the ancestor strain. ^8^Mutations: the mutational event that caused the changed repeat succession: del, deletion; pm, point-mutation; dupl, duplication. ^9^Repeat: the number of the repeat, which shows a point-mutation, which leads to a different repeat number and to a different *spa*-type. ^10^Nucleotide sequence: the changed nucleotide sequence of the repeat caused by one point-mutation, which is marked in bold.*

Multi-regression analyses of the different carriage profiles did not show any significant differences concerning gender, visits with exacerbations or lung function. Also, the carriage profiles of patients with prevalent and non-prevalent *spa-*types did not reveal any significant clinical differences.

### Age and Antibiotic Therapy Were Associated With Carriage Profiles

The more often patients were treated with antibiotics, the higher was the probability for the patients for being positive for only one *spa*-type (*p* = 0.005). Patients with dominant or related *spa*-types were significantly older (*p* = 0.026 and *p* = 0.006) compared to patients with non-dominant or unrelated *spa*-types.

### Whole Genome Sequence-Based Typing

**FIGURE 2:**
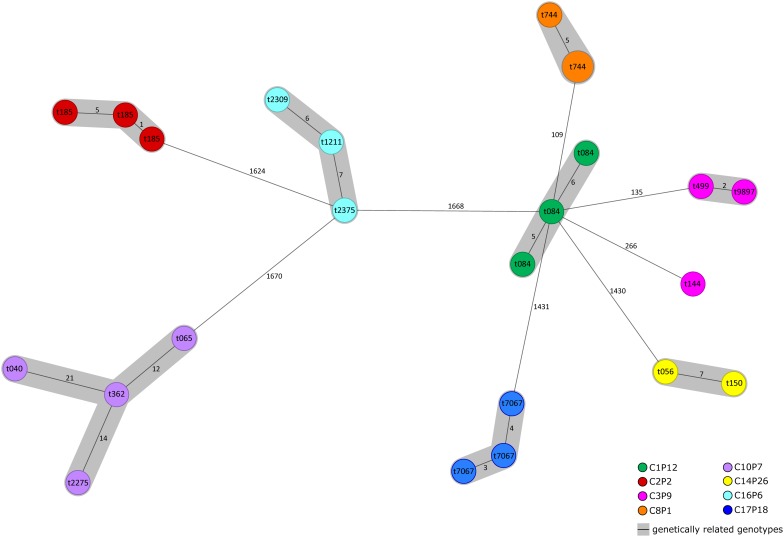
Minimum spanning tree of *S. aureus* isolates illustrating their genotypic relationship. 24 *S. aureus* strains are displayed representing different carriage profiles based on 1861 cgMLST target genes, pairwise ignoring missing values. Size of circles correlates with the number of identical genotypes. Different colors of circles indicate patients, from which *S. aureus* strains were chosen. *Spa*-types are given within different circles. Numbers on connecting lines indicate allele differences, gray shading indicates a close genetic similarity between different genotypes.

In total, 24 *S. aureus* strains were chosen from patients with different carriage profiles(Supplementary Table S5) for WGS-based analysis to determine their genetical relatedness. *In silico* extraction of *spa*-types resulted in the same *spa*-type as ascertained via classical *spa*-typing, except in one case, in which no *spa*-type could be detected via WGS. Minimum spanning tree analysis revealed eight clusters of genetical related isolates and one singleton (Figure 2). Each cluster contained *S. aureus* strains derived from only one patient, thereby confirming previous *spa*-typing analysis results. Only one isolate, categorized as related clone via classical *spa*-typing (t144, C3P9), was detected to be non-related to other isolates derived from the same patient, indicating either larger evolutionary events or co-infection with different clones of related *spa*-types.

### Susceptibility of *S. aureus* Isolates

The susceptibility testing of all isolates revealed low resistance rates for antistaphylococcal antibiotics (in percentage of tested isolates) with the following resistance rates: penicillin 74%, oxacillin (MRSA) 2%, erythromycin 27%, clindamycin 22%, gentamicin 8%, levofloxacin 3%, trimethoprim/sulfamethoxazole 9%, rifampin < 1%, vancomycin 0%, linezolid 0%, fusidic acid 1%, and fosfomycin 1%.

### Transmission of *S. aureus*

*Spa*-typing of all isolates allowed to observe, if transmission of *S. aureus* clones occurred within CF centers. If *spa*-types were cultured from at least three patients within one center, there could be a possible event of transmission, Supplementary Table S6. However, most of these *spa*-types belonged to prevalent clones (20 of 25 possible events). Therefore, the culture of these *spa*-types from several patients could be just due to the higher prevalence of such clones in the community. However, without knowledge about other epidemiological data, it is difficult to evaluate transmission. Also, in most patients the possible transmitted *spa-*types were not persistently cultured (Supplementary Table S4).

## Discussion

*Staphylococcus aureus* is one of the earliest and one of the most prevalent pathogens isolated from the airways of CF patients (Cystic Fibrosis Foundation Patient Registry, 2018; European Cystic Fibrosis Society Patient Registry, 2018), which persists for several years or even decades in spite of anti-staphylococcal therapy (Kahl et al., 1998, 2003; Andersen et al., 2014; Schwerdt et al., 2018). Nevertheless, there are only few studies, which evaluate the impact of *S. aureus* clonal lineages on lung disease in older CF patients (Wong et al., 2013; Junge et al., 2016).

Therefore, data from our earlier study (Junge et al., 2016) and from the analysis of *spa*-sequence typing of the collected isolates from this study are of interest to the CF community to shed more light on the behavior and impact of *S. aureus* and special *S. aureus* carriage profiles (prevalent, single, dominant, or related *spa*-types) during persistence in CF. Of importance is also that our study was conducted in an area, where CF patients are not treated with continuous anti-staphylococcal therapy as performed in the United Kingdom (Littlewood et al., 2009) or Australia (Bell and Robinson, 2008), but patients were rather treated, if *S. aureus* was cultured from the respiratory specimens or if symptoms occurred with *S. aureus* positive airway cultures.

Here, we determined the molecular clonality of almost 4000 *S. aureus* isolates from 183 CF patients during a 21-months study. There are some important findings of our study with the determination of *S. aureus* clonality during a long-term period in a large number of CF patients. Interestingly, there was not a special *S. aureus spa-*type, which we identified to be associated with a more severe lung disease during our study. Therefore, all *S. aureus* clones are able to cause lung disease and a more severe course of the CF disease may dependent on other most likely host related factors. Similar results have been shown recently in the study by Grundmann et al. (2010) who investigated the population structure of invasive *S. aureus* isolates. The study of invasive *S. aureus* revealed that all-cause mortality of *S. aureus* invasive infection was independent of *spa*-types indicating that there was no *spa*-type that stood out with respect to hypervirulence.

In our study, there were eight *S. aureus spa*-types that were isolated from more than 10 patients (prevalent clones). Interestingly, seven of the eight prevalent *spa-*types were not also the most prevalent *spa*-types of *S. aureus* isolates from studies of healthy nasal carriers in Germany [t084, t091, t008, t015 t012, t056, and t346; Holtfreter et al. (2016)], but all of our prevalent *spa*-types also belonged to the 20 most prevalent *spa*-types of invasive *S. aureus* isolates from a recent European study (Grundmann et al., 2010) indicating that most *S. aureus* isolates from CF patients originate from common clones present in the community setting and are not acquired during hospital contacts or stays. Furthermore, such prevalent *S. aureus* strains do not only belong to carriage strains (Holtfreter et al., 2016), but also to *S. aureus* strains that can cause severe life threatening infection (Grundmann et al., 2010). Also, in comparison to the study from Garbacz et al. (2018), in which 215 *S. aureus* isolates from 107 CF patients from Poland were characterized by *spa*-typing, four of our eight prevalent *spa*-types were also part of the five most common *spa-type*s of their study (t015, t084, t091, and t002). Therefore, our findings are representative for *S. aureus* isolates cultured also from the airways of CF patients from other countries.

We classified the *S. aureus* clones into four different *S. aureus* carriage groups according to the profiles of the cultivation of *S. aureus spa*-types throughout the study period in this long-term observational study as a *post hoc* analysis. Such grouping of patients according to *S. aureus* carriage profiles was used to compare the patients in regard to demographic and clinical findings.

The more patients were treated with antibiotics, the higher was the probability to culture only one *spa*-type (*p* = 0.005). The fact that antibiotic treatment affected the number of different clones, indicates that CF patients are highly susceptible for the acquisition of new *S. aureus* strains, if not treated with antibiotics. Such new incoming strains will be on the one hand in competition with residing strains, on the other hand, resident strains can acquire new genetic information by horizontal gene transfer (Quanjer et al., 2012) as shown in an earlier study by Langhanki et al. (2018). In consequence, the acquisition of genes could lead to an optimized gene pool, which could facilitate persistence.

Another significant finding of our study was that older patients were more likely to be culture positive for related *spa*-types, which share the overall composition of the repeats of the VNTR region of *spa*, but which are characterized by mutations in this region consisting of deletions of repeats, duplications, or point-mutations within repeats, all of which are leading to different *spa-type*s. The occurrence of related clones in CF patients has been shown earlier by our group in different studies (Kahl et al., 2003; Hirschhausen et al., 2013; Schwartbeck et al., 2016). To confirm the relatedness of clones not only by *spa-*typing, we also performed WGS of a number of isolates from different patients, Supplementary Table S5. Importantly, all isolates sequenced by WGS confirmed our *spa*-typing results except genome sequencing of *S. aureus* isolates from patient C3P9, of which two isolates were closely related but a third isolate differed by more than 200 bp indicating that either larger evolutionary events or co-infection with different clones of related *spa*-types occurred.

Interestingly, most mutations that occurred in the VNTR region were due to deletions of repeats, which is in line with Garofalo et al. (2012), who showed that there was an inverse correlation of the length of repeats and the length of *S. aureus* infection in CF patients and patients with chronic osteomyelitis. It has been shown that the VNTR region modulates the inflammatory response induced by protein A (Martin et al., 2009). Therefore, by deleting repeats during microevolution of the VNTR region, the pro-inflammatory response induced by protein A is decreased with less recruitment of neutrophils thereby facilitating *S. aureus* persistence in the hostile niche of CF airways.

There are some limitations of our study: with our 21-months study, we only got a short glimpse into the clonal behavior of *S. aureus* during persistence within the airways. Therefore, our data should be validated by long-term studies since in many patients, *S. aureus* persist for many years or even decades (Kahl et al., 1998; Hirschhausen et al., 2013; Andersen et al., 2014; Schwerdt et al., 2018). Another disadvantage was, that we only included patients, who were older than 6 years and who were already colonized or infected by *S. aureus* persistently. It would be also interesting to follow infants after neonatal screening to observe early *S. aureus* dynamics in CF patients.

## Conclusion

The molecular analysis of *S. aureus* during our prospective longitudinal observational study showed that transmission of clones within centers and antibiotic resistance rates of *S. aureus* were low. Furthermore, our study revealed that antibiotic therapy had a strong impact on *S. aureus* carriage profiles that were cultured from the airways. Patients that were more often treated were more likely to be positive for only one *S. aureus* clone indicating that antibiotic therapy prevented acquisition of other *S. aureus* clones thereby minimizing horizontal gene transfer by other new incoming clones. Furthermore, age had an impact not only on the culture of related but also on the culture of dominant clones. This indicates that during *S. aureus* persistence mutations in the VNTR region of *spa* are accumulating, especially such mutations, which cause a less pro-inflammatory response by protein A, and that clones, which are optimized for persistence in the airways, are being selected.

## Members of the Staphylococcal Cf Study Group

Department of Paediatric Pulmonology and Neonatology, Medizinische Hochschule Hannover, Hannover, Germany: Sibylle Junge. Clinical Research Group, Department of Paediatric Pulmonology and Neonatology, Medizinische Hochschule Hannover, Hannover, Germany: Burkhard Tümmler. CF-Center Innsbruck, Department of Paediatrics, University Hospital, Austria: Helmut Ellermunter. Department of Paediatrics, University Hospital Münster, Münster, Germany: Angelika Dübbers. Department of Paediatrics, Clemenshospital Münster, Münster, Germany: Peter Küster. Ruhr University Paediatric Clinic at St Josef Hospital, Bochum, Germany: Manfred Ballmann and Cordula Koerner-Rettberg. Department of Paediatrics, University Hospital Essen, Essen, Germany: Jörg Große-Onnebrink. Paediatricians “Kinderärztliche Ambulanz” Hamburg, Germany: Eberhardt Heuer and Wolfgang Sextro. CF Center, Department of Paediatrics, University Clinics Jena, Jena, Germany; Pediatric Pulmonology/Cystic Fibrosis, Brandenburg Medical School (MHB) University. Brandenburg an der Havel, Germany: Jochen G. Mainz. Department of Paediatrics, University Clinics Dresden, Dresden, Germany: Jutta Hammermann. Department of Pediatrics, University Clinics Tübingen, Tübingen, Germany: Ute Graepler-Mainka. Department of Paediatric Pulmonology and Immunology, Charité – Universitätsmedizin Berlin, Campus Virchow Klinikum, Berlin, Germany: Doris Staab. University Hospital Halle, Halle, Germany: Bettina Wollschläger. Children’s Hospital Osnabrück, Osnabrück, Germany: Rüdiger Szczepanski. Department of Paediatrics, University of Düsseldorf, Düsseldorf, Germany: Antje Schuster. Park Schönefeld Clinics Kassel, Germany: Friedrich-Karl Tegtmeyer. Ruhrlandklinik Essen, Germany: Sivagurunathan Sutharsan. University Clinics Leipzig, Germany: Alexandra Wald.

## Data Availability Statement

The raw data supporting the conclusions of this article will be made available by the authors, without undue reservation, to any qualified researcher.

## Ethics Statement

The studies involving human participants were reviewed and approved by the Ethik-Kommission der Ärztekammer Westfalen-Lippe und des Universitätsklinikum Münster (2007-496-f-S). Written informed consent to participate in this study was provided by the participants’ legal guardian/next of kin and by the participating patients.

## Author Contributions

CW analyzed the data and wrote the manuscript with the help of BK. DG analyzed the data. SK and AM were responsible for whole genome sequencing of *S. aureus* isolates, analysis of the sequencing data, and construction of the Figure 2. SH performed susceptibility analyses. NB and CH performed *spa*-typing. SJ, BT, HE, AD, PK, MB, CK-R, JG-O, EH, WS, JM, JH, UG-M, DS, BW, RS, AS, F-KT, SS, and AW provided patient specimens and CRFs. GP contributed to the study design. BK initiated and was responsible for the study and its design. All authors read and accepted the manuscript.

## Conflict of Interest

The authors declare that the research was conducted in the absence of any commercial or financial relationships that could be construed as a potential conflict of interest. The handling Editor declared a shared affiliation, though no other collaboration, with one of the authors JM at the time of review.

## Footnotes

1 https://www.spaserver.ridom.de/
