# Supplementary material for: Antibiotic Treatment and Age Are Associated With Staphylococcus aureus Carriage Profiles During Persistence in the Airways of Cystic Fibrosis Patients
Source: Front Microbiol. 2020 Feb 26;11:230. doi: 10.3389/fmicb.2020.00230 (PMC7055462; doi:10.3389/fmicb.2020.00230)
Supplement: Supplementary file 1 [file Table_1.docx]

**Table S1. Distribution of *spa*-types (clones) depending on the site.**

| **site** | **specimens** | **isolates** | ***spa* types** | **mean of *spa* types** |
| --- | --- | --- | --- | --- |
| **nose** | 753 | 1171 | 170 | 1.87 |

| **throat** | 782 | 1619 | 200 | 2.16 |
| --- | --- | --- | --- | --- |
| **sputum** | 394 | 1103 | 136 | 1.18 |

The number of *spa*-types within the different specimens is significantly different: p < 0.001.
