# Supplementary material for: Antibiotic Treatment and Age Are Associated With Staphylococcus aureus Carriage Profiles During Persistence in the Airways of Cystic Fibrosis Patients
Source: Front Microbiol. 2020 Feb 26;11:230. doi: 10.3389/fmicb.2020.00230 (PMC7055462; doi:10.3389/fmicb.2020.00230)
Supplement: Supplementary file 2 [file Table_2.docx]

**Table S2. *spa*-types, *spa* CCs and respective isolates in the 17 study centers.**

| **Center** | **Patients** | **Isolates** | ***spa*-types** | ***spa***  **clonal**  **complex^1^** | ***spa*-types within**  ***spa* CCs** | **no founder^2^** | **singletons^3^** | **excluded^4^** |
| --- | --- | --- | --- | --- | --- | --- | --- | --- |
| **1** | 15 | 462 | 50 | 6 | 23 | 4 | 19 | 4 |
| **2** | 12 | 262 | 35 | 3 | 15 | 8 | 10 | 2 |
| **3** | 10 | 304 | 38 | 4 | 20 | 4 | 11 | 0 |
| **4** | 4 | 258 | 13 | 1 | 4 | 2 | 7 | 0 |
| **5** | 22 | 355 | 40 | 3 | 12 | 12 | 13 | 3 |
| **6** | 4 | 96 | 14 | 1 | 4 | 4 | 5 | 1 |
| **7** | 6 | 147 | 21 | 0 | 0 | 6 | 12 | 3 |
| **8** | 12 | 190 | 29 | 2 | 8 | 6 | 13 | 2 |
| **9** | 4 | 73 | 14 | 0 | 0 | 4 | 10 | 0 |
| **10** | 7 | 94 | 16 | 2 | 6 | 2 | 7 | 1 |
| **11** | 13 | 239 | 34 | 6 | 21 | 2 | 11 | 0 |
| **12** | 10 | 170 | 35 | 3 | 13 | 10 | 11 | 1 |
| **13** | 6 | 121 | 17 | 2 | 11 | 2 | 4 | 0 |
| **14** | 25 | 407 | 53 | 5 | 18 | 16 | 14 | 5 |
| **15** | 3 | 66 | 12 | 0 | 0 | 4 | 8 | 0 |
| **16** | 7 | 202 | 32 | 3 | 12 | 9 | 10 | 1 |
| **17** | 23 | 447 | 48 | 6 | 25 | 10 | 10 | 3 |
| **all centers** | **183** | **3893** | **265** | **12** | **192** | **19** | **36** | **18** |

^1^*S. aureus* isolates were grouped into *spa* clonal complexes (*spa* CC) by BURP (based upon repeat pattern) analysis, which compares the base sequence of the repeat region of the individual *spa*-types

^2^for these *spa*-types the BURP algorithm did not detect a related *spa*-type in the investigated collection of isolates.

^3^without relation to any other *spa-*type in this study

^4^*spa-*types with 4 or less repeats were excluded from the analysis; The number of excluded *spa*-types is less than the sum, because some excluded *spa*-types were observed in different centers.
