# Supplementary material for: Antibiotic Treatment and Age Are Associated With Staphylococcus aureus Carriage Profiles During Persistence in the Airways of Cystic Fibrosis Patients
Source: Front Microbiol. 2020 Feb 26;11:230. doi: 10.3389/fmicb.2020.00230 (PMC7055462; doi:10.3389/fmicb.2020.00230)
Supplement: Supplementary file 4 [file Table_4.docx]

**Table S4. Mutational events in patients with related clones**

| **Patient** | **all clones^1^** | **related clones^2^** | **non-related^3^** | **% of related clones^4^** | **number of isolates^5^** | ***spa*-type^6^** | **VNTR region^7^** | **mutations^8^** | **repeat^9^** | **nucleotide sequence of the repeat region^10^** |
| --- | --- | --- | --- | --- | --- | --- | --- | --- | --- | --- |
| C3P12 | 11 | 4 | 7 | 36% | 29 | **t008** | 11-**19**-12-21-17-34-24-34-22-**25** |  |  |  |
|  |  |  |  |  | 12 | t024 | 11-12-21-17-34-24-34-22-25 | del | r24 | AAAGAAGATGGCAACAA**G**CCTGGT |
|  |  |  |  |  | 1 | t1709 | 11-19-12-21-17-34-24-34-22-**24** | pm | r25 | AAAGAAGATGGCAACAA**A**CCTGGT |
|  |  |  |  |  | 1 | t681 | 11-19-12-21-17-**17**-34-24-34-22-25 | dupl |  |  |
|  |  |  |  |  |  |  |  |  |  |  |
| C4P1 | 3 | 2 | 1 | 67% | 58 | **t012** | 15-12-16-02-16-02-25-17-24-24 |  |  |  |
|  |  |  |  |  | 6 | t018 | 15-12-16-02-16-02-25-17-24-24-**24** | dupl |  |  |
|  |  |  |  |  |  |  |  |  |  |  |
| C4P4 | 4 | 2 | 2 | 50% | 2 | **t950** | 08-16-34-**17-34**-16-34 |  |  |  |
|  |  |  |  |  | 1 | t550 | 08-17-34-16-34 | del |  |  |
|  |  |  |  |  |  |  |  |  |  |  |
| C5P7 | 2 | 2 | 0 | 100% | 11 | **t015** | 08-16-02-16-34-13-17-34-16-34 |  |  |  |
|  |  |  |  |  | 3 | t1203 | 08-16-02-16-34-13-17-**17**-34-16-34 | dupl |  |  |
|  |  |  |  |  |  |  |  |  |  |  |
| C6P3 | 3 | 2 | 1 | 67% | 10 | **t005** | 26-23-13-23-31-05-17-**25**-17-25-16-28 |  |  |  |
|  |  |  |  |  | 12 | t006 | 26-23-13-23-31-05-17-17-25-16-28 | del |  |  |
|  |  |  |  |  |  |  |  |  |  |  |
|  |  |  |  |  |  |  |  |  |  |  |
| C9P1 | 4 | 2 | 2 | 50% | 2 | **t330** | 09-02-16-34-34-17-34-**16**-34 |  |  |  |
|  |  |  |  |  | 1 | t5775 | 09-02-16-34-34-17-34-34 | del |  |  |
|  |  |  |  |  |  |  |  |  |  |  |
| C10P4 | 2 | 2 | 0 | 100% | 11 | **t166** | 04-44-33-31-12-16-34-16-12-25-22-34 |  | r44 | AAAGAAGACAACAAGCCTGG**T** |
|  |  |  |  |  | 1 | t352 | 04-54-31-12-16-34-16-12-25-22-34 | del and pm | r54 | AAAGAAGACAACAAGCCTGG**C** |
|  |  |  |  |  |  |  |  |  |  |  |
|  |  |  |  |  |  |  |  |  |  |  |
| C10P7 | 5 | 3 | 2 | 60% | 12 | **t065** | 09-02-16-**34**-13-17-**34**-16-34 |  | r34 | AAAGAAGACAACAA**A**AAACCTGGT |
|  |  |  |  |  | 1 | t2275 | 09-02-16-34-13-17-**13**-16-34 | pm | r13 | AAAGAAGACAACAA**C**AAACCTGGT |
|  |  |  |  |  | 1 | t040 | 09-02-16-13-17-34-16-34 | del |  |  |
|  |  |  |  |  |  |  |  |  |  |  |
| C11P6 | 5 | 3 | 2 | 60% | 27 | **t159** | 14-44-13-12-**17**-17-**23**-18-17 |  | r82 | AAAGAAGAC**A**GCAACAAACCTGGC |
|  |  |  |  |  | 1 | t6763 | 14-44-13-12-17-17-**82**-18-17 | pm | r23 | AAAGAAGAC**G**GCAACAAACCTGGC |
|  |  |  |  |  | 1 | t645 | 14-44-13-12-17-23-18-17 | del |  |  |
|  |  |  |  |  |  |  |  |  |  |  |
| C11P9 | 3 | 2 | 1 | 67% | 14 | **t008** | 11-19-12-21-17-34-24-34-22-25 |  |  |  |
|  |  |  |  |  | 1 | t068 | 11-**19**-19-12-21-17-34-24-34-22-25 | dupl |  |  |
|  |  |  |  |  |  |  |  |  |  |  |
| C11P13 | 2 | 2 | 0 | 100% | 20 | **t505** | 08-02-16-**34**-13-17-34-16-34 |  | r34 | AAAGAAGACAACAA**A**AAACCTGGT |
|  |  |  |  |  | 1 | t466 | 08-02-16-**13**-13-17-34-16-34 | pm | r13 | AAAGAAGACAACAA**C**AAACCTGGT |
|  |  |  |  |  |  |  |  |  |  |  |
| C12P3 | 4 | 2 | 2 | 50% | 10 | **t091** | 07-23-21-17-34-**12**-23-02-12-23 |  | r12 | AAAGAAGACA**A**CAACAAGCCTGGT |
|  |  |  |  |  | 1 | t1689 | 07-23-21-17-34-**66**-23-02-12-23 | pm | r66 | AAAGAAGACA**G**CAACAAGCCTGGT |
|  |  |  |  |  |  |  |  |  |  |  |
| C13P3 | 4 | 3 | 1 | 75% | 18 | **t346** | 07-23-12-34-12-12-23-02-12-23 |  |  |  |
|  |  |  |  |  | 8 | t084 | 07-23-12-34-**34**-12-12-23-02-12-23 | dupl | r12 | AAAGAAGACA**A**CAACAAGCCTGGT |
|  |  |  |  |  | 3 | t2398 | 07-23-12-34-12-66-23-02-12-23 | pm | r66 | AAAGAAGACA**G**CAACAAGCCTGGT |
|  |  |  |  |  |  |  |  |  |  |  |
| C13P5 | 11 | 6 | 5 | 54% | 25 | **t002** | 26-23-17-**34-17**-**20**-17-12-17-**16** |  |  |  |
|  |  |  |  |  | 8 | t509 | 26-23-17-20-17-12-**17-16** | del |  |  |
|  |  |  |  |  | 2 | t5686 | 26-23-17-20-17-12 | del | r20 | AAAGAAGACA**A**CAACAAACCTGGC |
|  |  |  |  |  | 1 | t3012 | 26-23-23-17-34-20-17-12-17-16 | dupl | r82 | AAAGAAGACA**G**CAACAAACCTGGC |
|  |  |  |  |  | 6 | t2164 | 26-23-17-34-17-**82**-17-12-17-16 | pm | r16 | AAAGAAGACGGCAACAA**A**CCTGGT |
|  |  |  |  |  | 1 | t686 | 26-23-17-34-17-20-17-12-17-17 | pm | r17 | AAAGAAGACGGCAACAA**G**CCTGGT |
|  |  |  |  |  |  |  |  |  |  |  |
| C14P2 | 3 | 2 | 1 | 67% | 19 | **t166** | 04-**44**-33-31-12-16-34-16-12-25-22-34 |  |  |  |
|  |  |  |  |  | 2 | t1057 | 04-33-31-12-16-34-16-12-25-22-34 | del |  |  |
|  |  |  |  |  |  |  |  |  |  |  |
| C14P4 | 3 | 2 | 1 | 67% | 6 | **t015** | 08-16-02-16-34-13-17-34-16-34 |  |  |  |
|  |  |  |  |  | 1 | t1574 | 08-16-02-16-34-**13-**13-17-34-16-34 | dupl |  |  |
|  |  |  |  |  |  |  |  |  |  |  |
| C14P6 | 4 | 2 | 2 | 50% | 16 | **t084** | 07-23-12-34-34-12-**12**-23-02-12-23 |  |  |  |
|  |  |  |  |  | 2 | t085 | 07-23-12-34-34-12-23-02-12-23 | del |  |  |
|  |  |  |  |  |  |  |  |  |  |  |
| C14P12 | 2 | 2 | 0 | 100% | 16 | **t676** | 08-12-16-02-17-16 |  |  |  |
|  |  |  |  |  | 1 | t12678 | 08-12-16-02-17-16-16 | dupl |  |  |
|  |  |  |  |  |  |  |  |  |  |  |
| C14P26 | 4 | 2 | 2 | 50% | 11 | **t056** | 04-20-12-17-20-17-12-17-**17** |  |  |  |
|  |  |  |  |  | 1 | t150 | 04-20-12-17-20-17-12-17 | del |  |  |
|  |  |  |  |  |  |  |  |  |  |  |
| C14P30 | 3 | 2 | 1 | 67% | 4 | t**7064** | 07-23-12-34-34-12-12-23-02-02-**02**-12-23 |  |  |  |
|  |  |  |  |  | 6 | t144 | 07-23-12-34-34-12-12-23-02-02-12-23 | del |  |  |
|  |  |  |  |  |  |  |  |  |  |  |
| C16P2 | 4 | 2 | 2 | 50% | 7 | **t003** | 26-17-20-17-12-17-**17**-16 |  |  |  |
|  |  |  |  |  | 2 | t045 | 26-17-20-17-12-17-16 | del |  |  |
|  |  |  |  |  |  |  |  |  |  |  |
| C16P6 | 6 | 3 | 3 | 50% | 21 | **t1211** | 08-13-17-17-**17**-23-18-17 |  |  |  |
|  |  |  |  |  | 1 | t2309 | 08-13-17-17-17-17-23-18-17 | dupl |  |  |
|  |  |  |  |  | 1 | t2375 | 08-13-17-17-23-18-17 | del |  |  |
|  |  |  |  |  |  |  |  |  |  |  |
| C16P8 | 5 | 5 | 0 | 100% | 47 | **t084** | 07-23-12-34-**34**-**12**-12-**23**-**02**-12-23 |  |  |  |
|  |  |  |  |  | 8 | t346 | 07-23-12-34-12-12-23-02-12-23 | del | r2 | AAAGAAGACAACAAAAAACCTGG**C** |
|  |  |  |  |  | 1 | t7272 | 07-23-12-34-34-12-12-23-34-12-23 | pm | r34 | AAAGAAGACAACAAAAAACCTGG**T** |
|  |  |  |  |  | 1 | t8027 | 07-23-12-34-34-12-12-12-02-12-23 | del and dupl |  |  |
|  |  |  |  |  | 1 | t1492 | 07-23-12-34-34-34-12-23-02-12-23 | del and dupl |  |  |
|  |  |  |  |  |  |  |  |  |  |  |
| C17P1 | 4 | 2 | 2 | 50% | 11 | **t9884** | 15-12-16-**16**-02-**06**-16-02-25-17-24 |  |  |  |
|  |  |  |  |  | 2 | t021 | 15-12-16-02-16-02-25-17-24 | del |  |  |
|  |  |  |  |  |  |  |  |  |  |  |
| C17P6 | 4 | 2 | 2 | 50% | 5 | **t284** | 14-44-13-17-17-23-18-17 |  |  |  |
|  |  |  |  |  | 1 | t159 | 14-44-13-12-17-17-23-18-17 | dupl |  |  |
|  |  |  |  |  |  |  |  |  |  |  |
| C17P8 | 2 | 2 | 0 | 100% | 24 | **t084** | 07-23-12-34-34-12-**12**-23-02-12-23 |  |  |  |
|  |  |  |  |  | 1 | t774 | 07-23-12-34-34-12-12-12-23-02-12-23 | dupl |  |  |
|  |  |  |  |  |  |  |  |  |  |  |
| C17P12 | 4 | 2 | 2 | 50% | 12 | **t258** | 04-21-12-41-20-17-**12**-12-12-17 |  |  |  |
|  |  |  |  |  | 6 | t1671 | 04-21-12-41-20-17-12-12-12-12-17 | dupl |  |  |
|  |  |  |  |  |  |  |  |  |  |  |
| z17p13 | 6 | 4 | 2 | 67% | 23 | **t514** | 04-20-12-17-20-17-12-17-17-**17** |  |  |  |
|  |  |  |  |  | 5 | t9894 | 04-20-12-17-20-17-12-17-17-17-17 | dupl |  |  |
|  |  |  |  |  | 2 | **t159** | 14-44-**13**-12-17-17-23-18-17 |  |  |  |
|  |  |  |  |  | 1 | t162 | 14-44-12-17-17-23-18-17 | del |  |  |
|  |  |  |  |  |  |  |  |  |  |  |
| C17P14 | 3 | 2 | 1 | 67% | 15 | **t012** | 15-12-16-**02**-**16**-02-25-17-24-24 |  | r02 | AAAGAAGACAACAAAAAACCTGG**C** |
|  |  |  |  |  | 2 | t1070 | 15-12-16-**34**-02-25-17-24-24 | del and pm | r34 | AAAGAAGACAACAAAAAACCTGG**T** |
|  |  |  |  |  |  |  |  |  |  |  |
| C17P16 | 2 | 2 | 0 | 100% | 20 | **t127** | 07-23-21-16-34-33-13 |  |  |  |
|  |  |  |  |  | 12 | t591 | 07-23-**21**-21-16-34-33-13 | dupl |  |  |
|  |  |  |  |  |  |  |  |  |  |  |
| C17P17 | 4 | 2 | 2 | 50% | 12 | **t091** | 07-23-21-17-34-12-23-02-12-23 |  | r12 | AAAGAAGACA**A**CAACAAGCCTGGT |
|  |  |  |  |  | 1 | t1689 | 07-23-21-17-34-66-23-02-12-23 | pm | r66 | AAAGAAGACA**G**CAACAAGCCTGGT |
|  |  |  |  |  |  |  |  |  |  |  |
| C17P20 | 2 | 2 | 0 | 100% | 27 | **t2439** | 26-23-13-23-31-05-17-**25**-25-17-25-16-28 |  |  |  |
|  |  |  |  |  | 1 | t005 | 26-23-13-23-31-05-17-25-17-25-16-28 | del |  |  |
|  |  |  |  |  |  |  |  |  |  |  |
| C17P23 | 2 | 2 | 0 | 100% | 27 | **t2509** | 15-**21**-12-16-02-16-02-25-17-24 |  |  |  |
|  |  |  |  |  | 1 | t021 | 15-12-16-02-16-02-25-17-24 | del |  |  |

^1^all clones: All different *spa-*types isolated from the airways of this patient.

^2^related clones: Number of *spa*-types, which evolved most likely due to mutational events in the "variable number of repeat" region of spa during persistence.

^3^non-related clones: Number of additional clones with *spa*-types characterized by a non-related repeat region of *spa.*

^4^percentage of related clones: Percentage of isolates with related *spa*-types.

^5^number of isolates with the respective *spa*-types

^6^*spa*-type: The different *spa*-types of patients with related *spa*-types; anchestor strains are marked in bold.

^7^VNTR-region: The sequence of the repeats within the VNTR-region; the mutated repeats are marked in bold in the anchestor strain.

^8^mutations: The mutational event that caused the changed repeat succession: del- deletion; pm - poin-mutation; dupl - duplication.

^9^repeat: The number of the repeat, which shows a point-mutation, which leads to a different repeat number and to a different *spa*-type.

^10^nucleotide sequence: The changed nucleotide sequence of the repeat caused by one point-mutation, which is marked in bold.
