# Supplementary material for: Antibiotic Treatment and Age Are Associated With Staphylococcus aureus Carriage Profiles During Persistence in the Airways of Cystic Fibrosis Patients
Source: Front Microbiol. 2020 Feb 26;11:230. doi: 10.3389/fmicb.2020.00230 (PMC7055462; doi:10.3389/fmicb.2020.00230)
Supplement: Supplementary file 5 [file Table_5.docx]

**Table S5. Isolates for whole genome sequencing**

|  | **sequencing nr.** | **Isolate** | **Center** | **Patient** | **site** | **spa-type** | **repeats** | **carriage profile** |
| --- | --- | --- | --- | --- | --- | --- | --- | --- |
| C**1P12** | **16** | S39 1 | 1 | 12 | sputum | t084 |  | prevalent clone |
|  | **15** | S490 1 | 1 | 12 | sputum | t084 |  | prevalent clone |
|  | **23** | S1482 2 | 1 | 12 | nose | t084 |  | prevalent clone |
| C**2P2** | **18** | S65 2 | 2 | 2 | throat | t185 |  | non-prevalent clone |
|  | **17** | S154 4 | 2 | 2 | sputum | t185 |  | non-prevalent clone |
|  | **14** | S2477 | 2 | 2 | throat | t185 |  | non-prevalent clone |
| C**17P18** | **12** | S1703 | 17 | 18 | sputum | t7067 |  | only one clone |
|  | **13** | S2917 3 | 17 | 18 | sputum | t7067 |  | only one clone |
|  | **21** | S3616 | 17 | 18 | sputum | t7067 |  | only one clone |
| C**8P1** | **22** | S101 | 8 | 1 | throat | t774 |  | dominant clone |
|  | **20** | S795 1 | 8 | 1 | throat | t774 |  | dominant clone |
|  | **24** | S2208 2 | 8 | 1 | throat | t774 |  | dominant clone |
| C**3P9** | **5** | S1726 | 3 | 9 | sputum | t499 | 07-23-12-12-34-12-12-23-02-12-23 | related clones |
|  | **8** | S2640 1 | 3 | 9 | nose | t144 | 07-23-12-34-34-12-12-23-02-02-12-23 | related clones |
|  | **7** | S2639 2 | 3 | 9 | sputum | t9897 | 07-23-12-12-34-12-12-12-23-02-12-23 | related clones |
| C**10P7** | **19** | S705 1 | 10 | 7 | throat | t065 | 09-02-16-34-13-17-34-16-34 | related clones |
|  | **6** | S2892 4 | 10 | 7 | throat | t2275 | 09-02-16-34-13-17-13-16-34 | related clones |
|  | **1** | S3690 1 | 10 | 7 | throat | t362 | 09-34 | related clones |
|  | **2** | S3751 3 | 10 | 7 | throat | t040 | 09-02-16-13-17-34-16-34 | related clones |
| C**14P26** | **10** | S2618 2 | 14 | 26 | throat | t056 | 04-20-12-17-20-17-12-17-17 | related clones |
|  | **9** | S2618 6 | 14 | 26 | throat | t150 | 04-20-12-17-20-17-12-17 | related clones |
| C**16P6** | **3** | S1201 1 | 16 | 6 | sputum | t1211 | 08-13-17-17-17-23-18-17 | related clones |
|  | **11** | S1804 2 | 16 | 6 | sputum | t2309 | 08-13-17-17-17-17-23-18-17 | related clones |
|  | **4** | S3450 2 | 16 | 6 | nose | t2375 | 08-13-17-17-23-18-17 | related clones |
|  |  |  |  |  |  |  |  |  |
