# Supplementary material for: Antibiotic Treatment and Age Are Associated With Staphylococcus aureus Carriage Profiles During Persistence in the Airways of Cystic Fibrosis Patients
Source: Front Microbiol. 2020 Feb 26;11:230. doi: 10.3389/fmicb.2020.00230 (PMC7055462; doi:10.3389/fmicb.2020.00230)
Supplement: Supplementary file 6 [file Table_6.docx]

**Table S6. Possible transmission of *S. aureus* *spa*-types within CF centers**

| **center** | ***spa-*type** | **patients with *spa*-type** | **persistent in**  **patients** |
| --- | --- | --- | --- |
| 1 | t008 | 5 | 2 |
| 1 | t084 | 4 | 1 |
| 1 | **t1050^1^** | 3 | 2 |
| 1 | t091 | 4 | 1 |
| 1 | **t166** | 3 | 1 |
| 2 | t084 | 3 | 3 |
| 2 | t346 | 3 | 0 |
| 2 | **t548** | 3 | 3 |
| 3 | t084 | 3 | 0 |
| 3 | **t499** | 3 | 1 |
| 5 | t002 | 3 | 3 |
| 5 | t008 | 4 | 2 |
| 5 | t084 | 3 | 1 |
| 7 | t091 | 3 | 0 |
| 8 | t084 | 3 | 1 |
| 11 | t012 | 3 | 1 |
| 11 | t056 | 3 | 1 |
| 11 | t091 | 3 | 2 |
| 13 | t346 | 3 | 1 |
| 14 | t015 | 3 | 0 |
| 14 | t084 | 3 | 0 |
| 14 | t091 | 3 | 1 |
| 14 | **t127** | 3 | 1 |
| 17 | t012 | 4 | 2 |
| 17 | t084 | 3 | 1 |

^1^not prevalent *spa-*types are shown in bold
