# Supplementary material for: Antibiotic Treatment and Age Are Associated With Staphylococcus aureus Carriage Profiles During Persistence in the Airways of Cystic Fibrosis Patients
Source: Front Microbiol. 2020 Feb 26;11:230. doi: 10.3389/fmicb.2020.00230 (PMC7055462; doi:10.3389/fmicb.2020.00230)
Supplement: Supplementary file 8 [file Presentation_1.PPTX]

## Slide 1
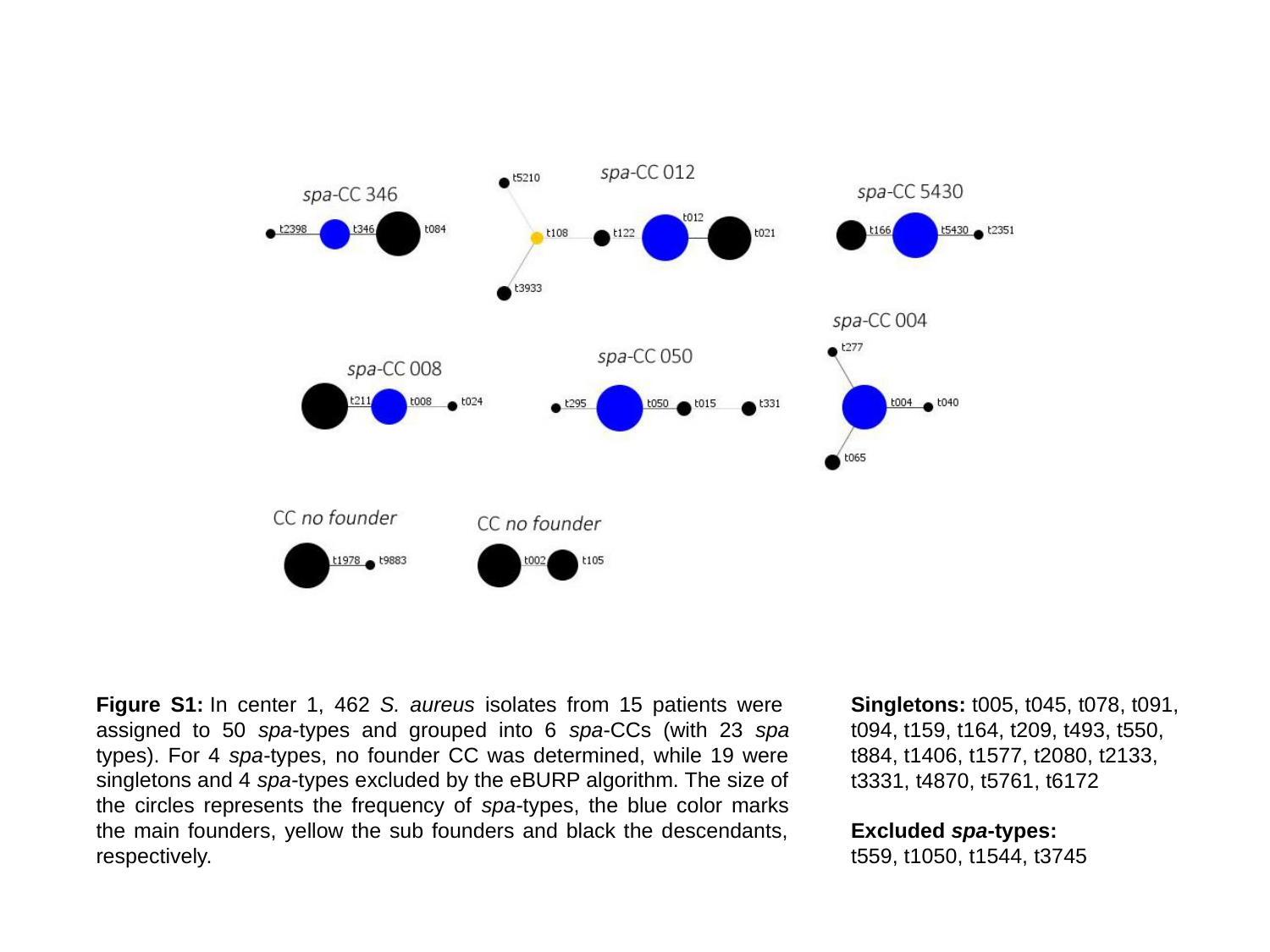

Singletons: t005, t045, t078, t091,
t094, t159, t164, t209, t493, t550,
t884, t1406, t1577, t2080, t2133,
t3331, t4870, t5761, t6172
Excluded spa-types:
t559, t1050, t1544, t3745
Figure S1: In center 1, 462 S. aureus isolates from 15 patients were  assigned to 50 spa-types and grouped into 6 spa-CCs (with 23 spa types). For 4 spa-types, no founder CC was determined, while 19 were singletons and 4 spa-types excluded by the eBURP algorithm. The size of the circles represents the frequency of spa-types, the blue color marks the main founders, yellow the sub founders and black the descendants, respectively.

## Slide 2
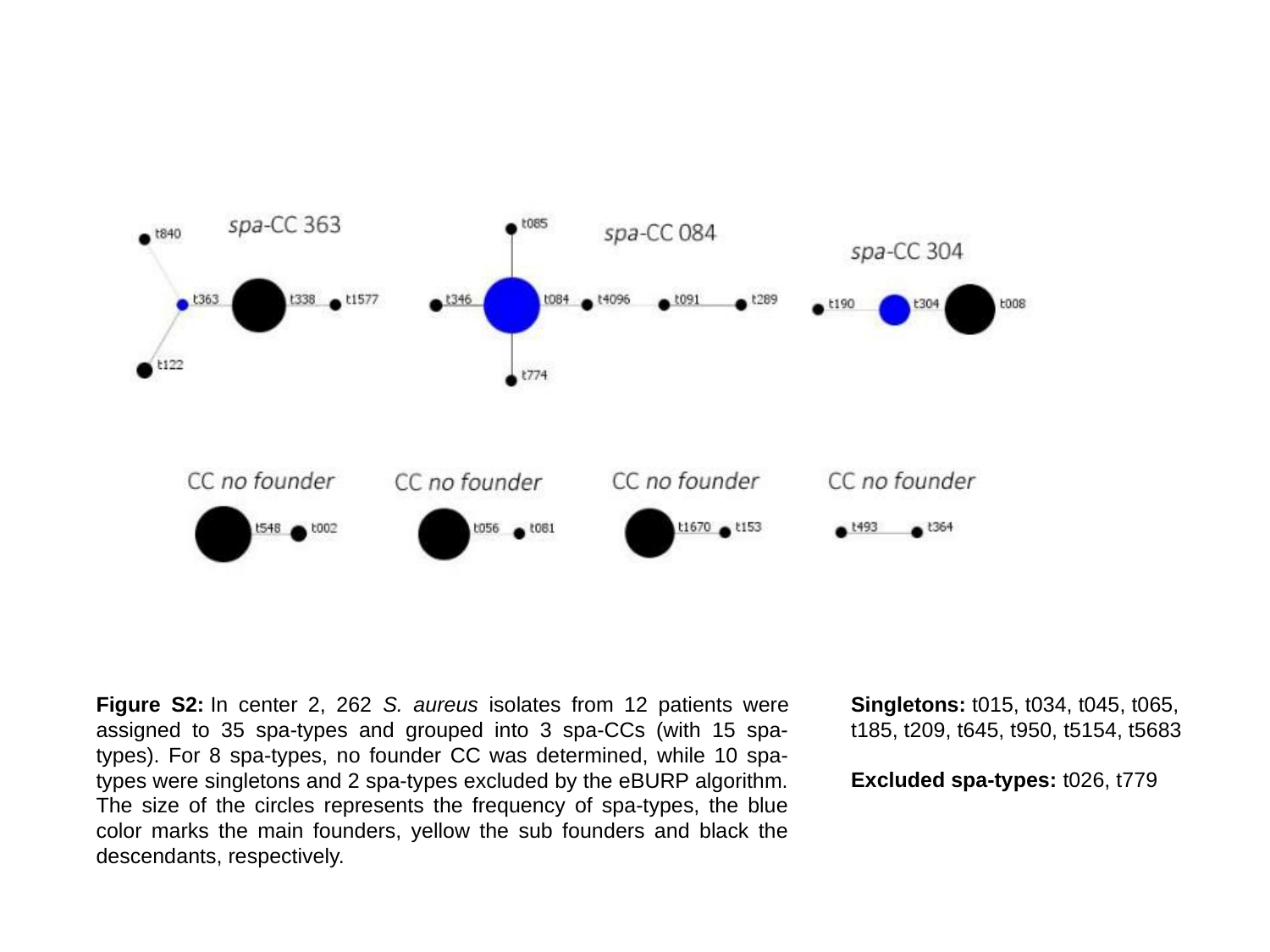

Singletons: t015, t034, t045, t065,
t185, t209, t645, t950, t5154, t5683
Excluded spa-types: t026, t779
Figure S2: In center 2, 262 S. aureus isolates from 12 patients were assigned to 35 spa-types and grouped into 3 spa-CCs (with 15 spa-types). For 8 spa-types, no founder CC was determined, while 10 spa-types were singletons and 2 spa-types excluded by the eBURP algorithm. The size of the circles represents the frequency of spa-types, the blue color marks the main founders, yellow the sub founders and black the descendants, respectively.

## Slide 3
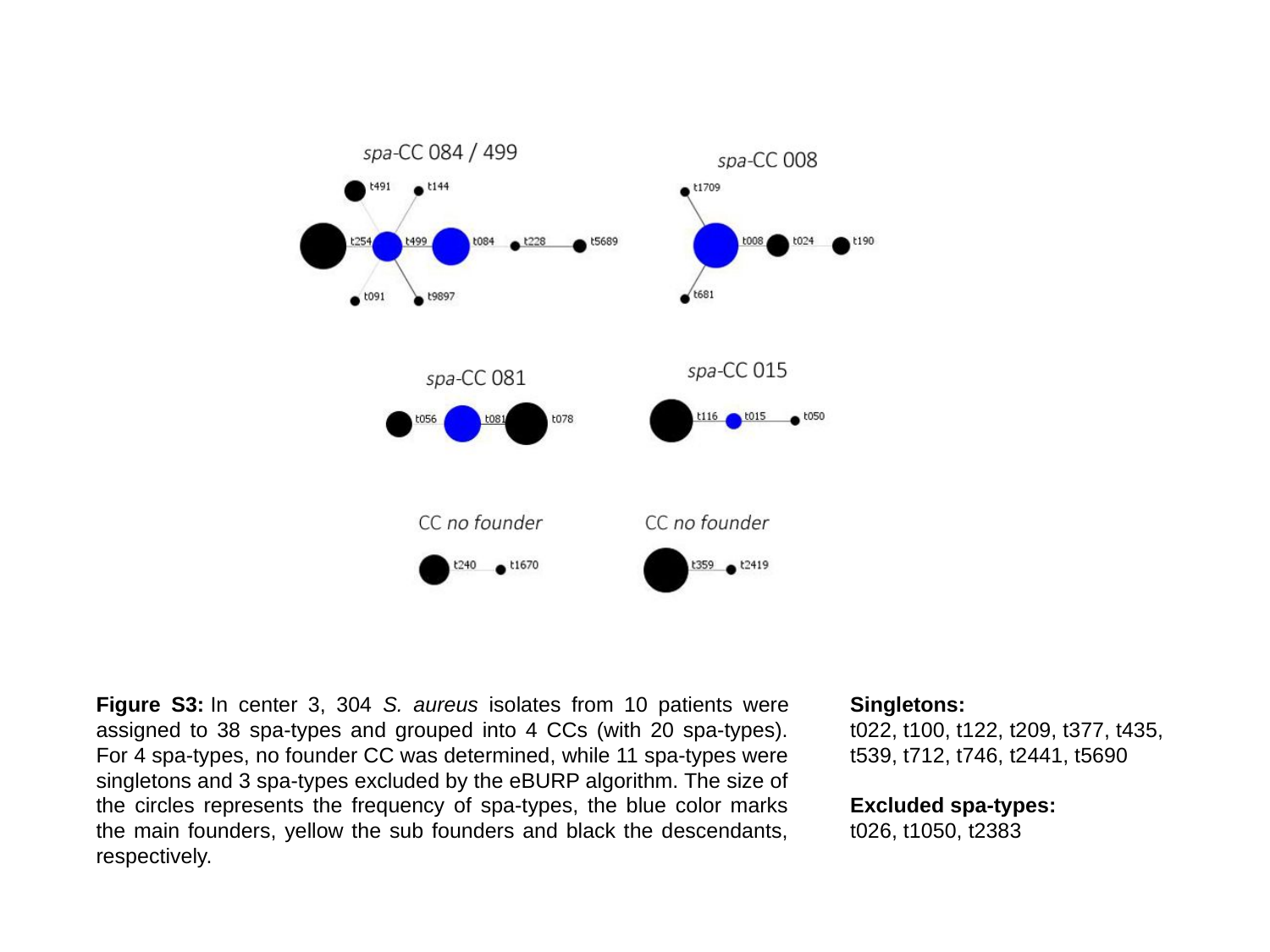

Singletons:
t022, t100, t122, t209, t377, t435,
t539, t712, t746, t2441, t5690
Excluded spa-types: 
t026, t1050, t2383
Figure S3: In center 3, 304 S. aureus isolates from 10 patients were assigned to 38 spa-types and grouped into 4 CCs (with 20 spa-types). For 4 spa-types, no founder CC was determined, while 11 spa-types were singletons and 3 spa-types excluded by the eBURP algorithm. The size of the circles represents the frequency of spa-types, the blue color marks the main founders, yellow the sub founders and black the descendants, respectively.

## Slide 4
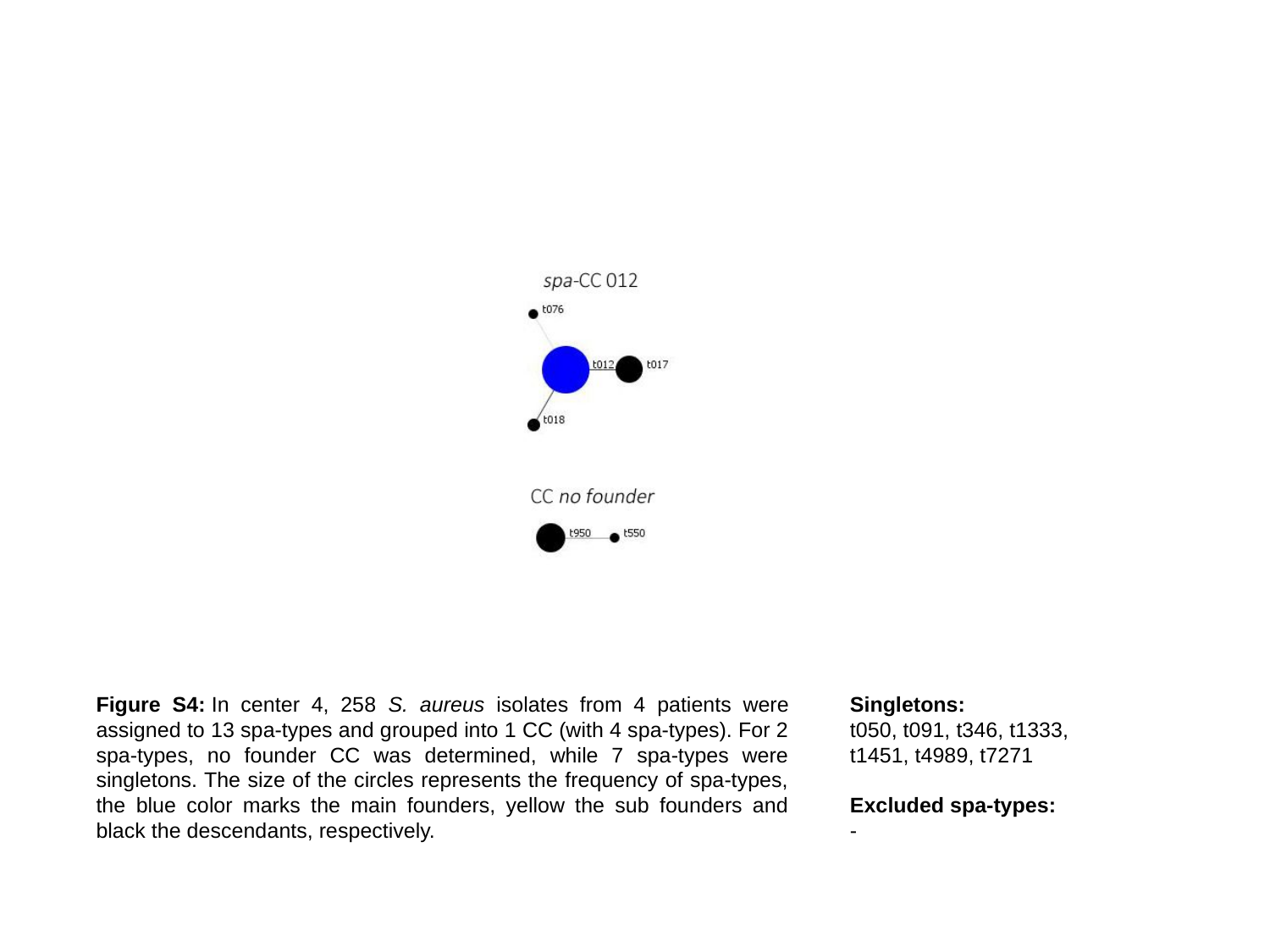

Singletons:
t050, t091, t346, t1333,
t1451, t4989, t7271
Excluded spa-types:
-
Figure S4: In center 4, 258 S. aureus isolates from 4 patients were assigned to 13 spa-types and grouped into 1 CC (with 4 spa-types). For 2 spa-types, no founder CC was determined, while 7 spa-types were singletons. The size of the circles represents the frequency of spa-types, the blue color marks the main founders, yellow the sub founders and black the descendants, respectively.

## Slide 5
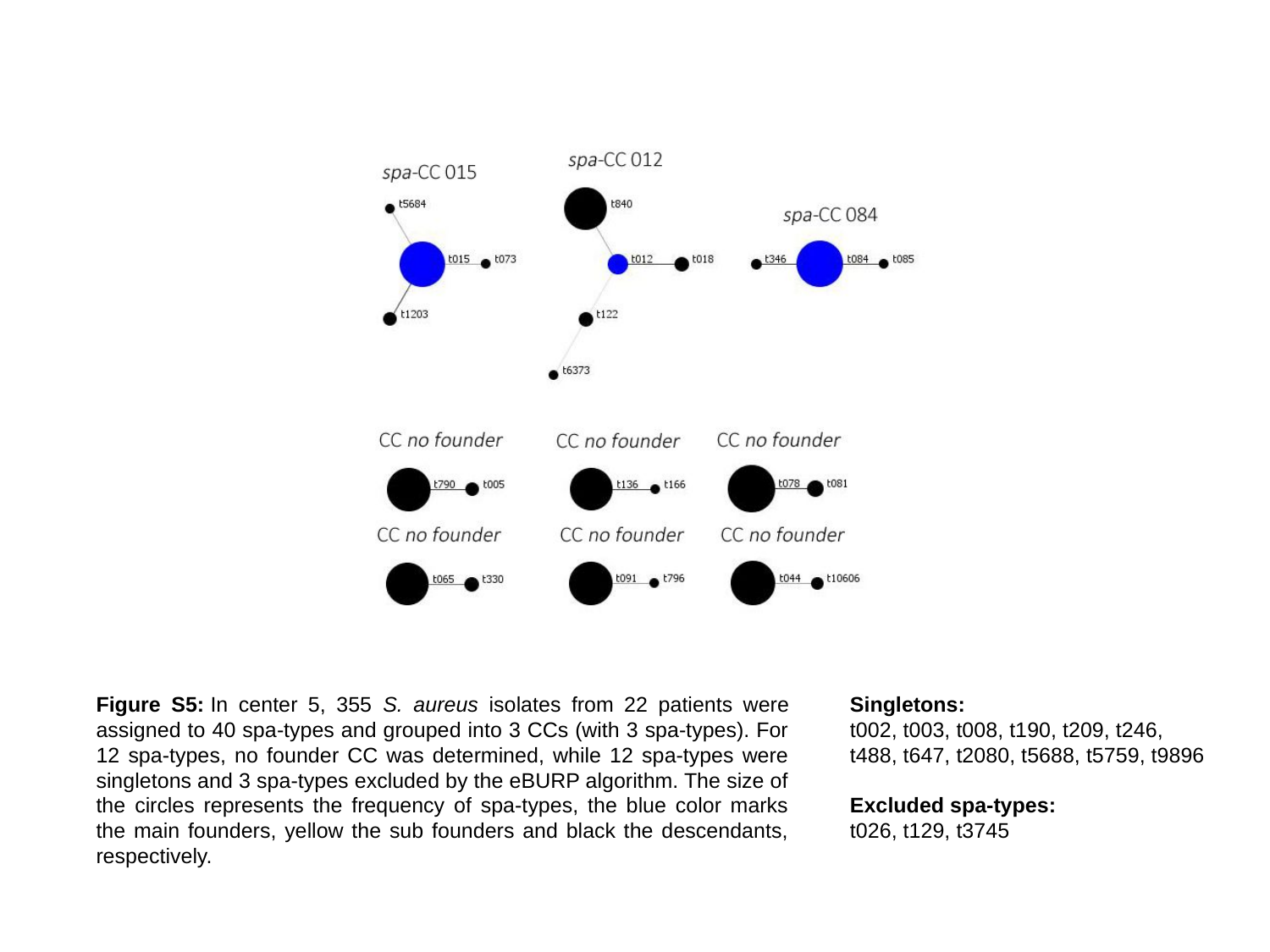

Singletons:
t002, t003, t008, t190, t209, t246,
t488, t647, t2080, t5688, t5759, t9896
Excluded spa-types:
t026, t129, t3745
Figure S5: In center 5, 355 S. aureus isolates from 22 patients were assigned to 40 spa-types and grouped into 3 CCs (with 3 spa-types). For 12 spa-types, no founder CC was determined, while 12 spa-types were singletons and 3 spa-types excluded by the eBURP algorithm. The size of the circles represents the frequency of spa-types, the blue color marks the main founders, yellow the sub founders and black the descendants, respectively.

## Slide 6
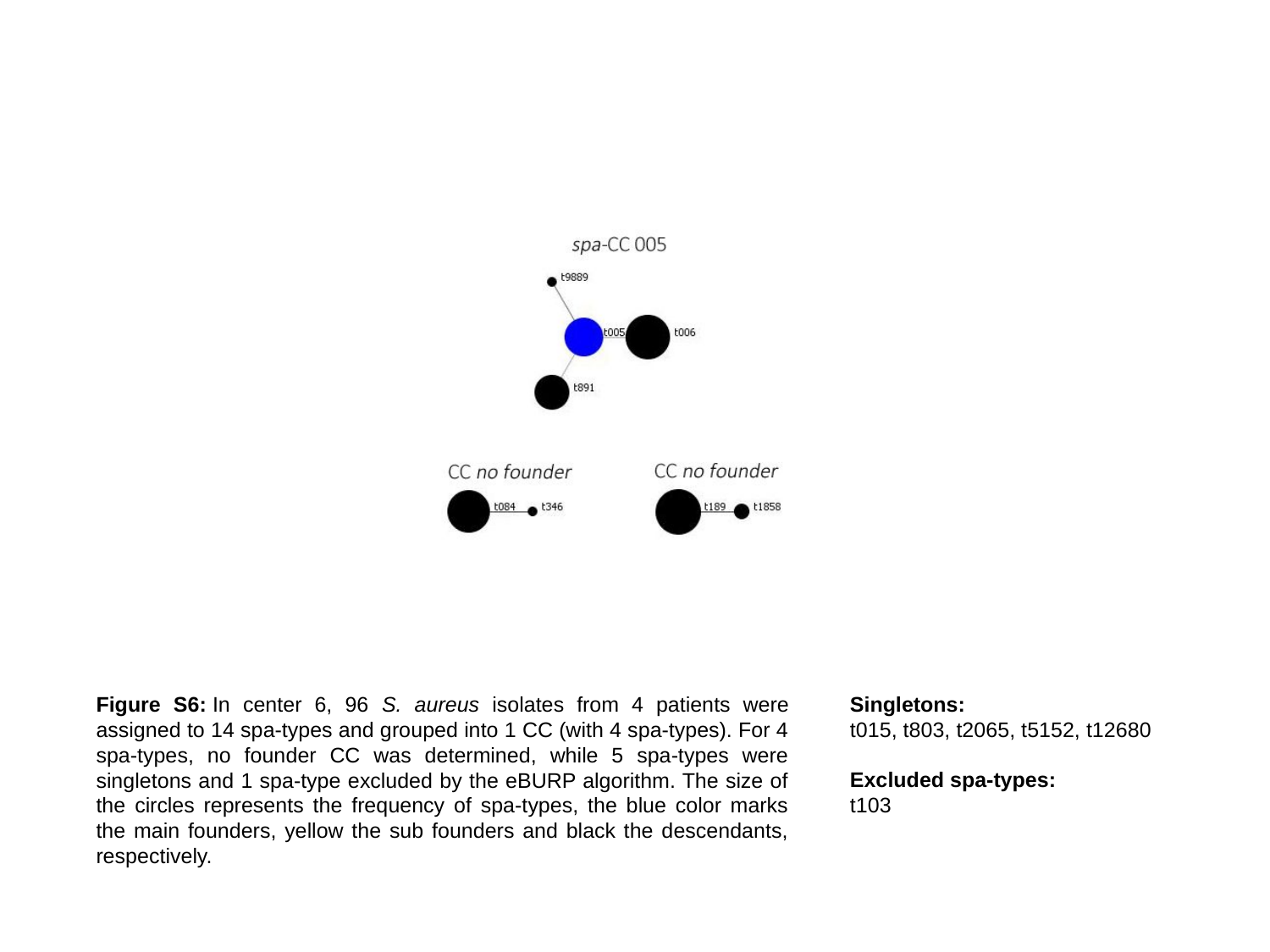

Singletons:
t015, t803, t2065, t5152, t12680
Excluded spa-types:
t103
Figure S6: In center 6, 96 S. aureus isolates from 4 patients were assigned to 14 spa-types and grouped into 1 CC (with 4 spa-types). For 4 spa-types, no founder CC was determined, while 5 spa-types were singletons and 1 spa-type excluded by the eBURP algorithm. The size of the circles represents the frequency of spa-types, the blue color marks the main founders, yellow the sub founders and black the descendants, respectively.

## Slide 7
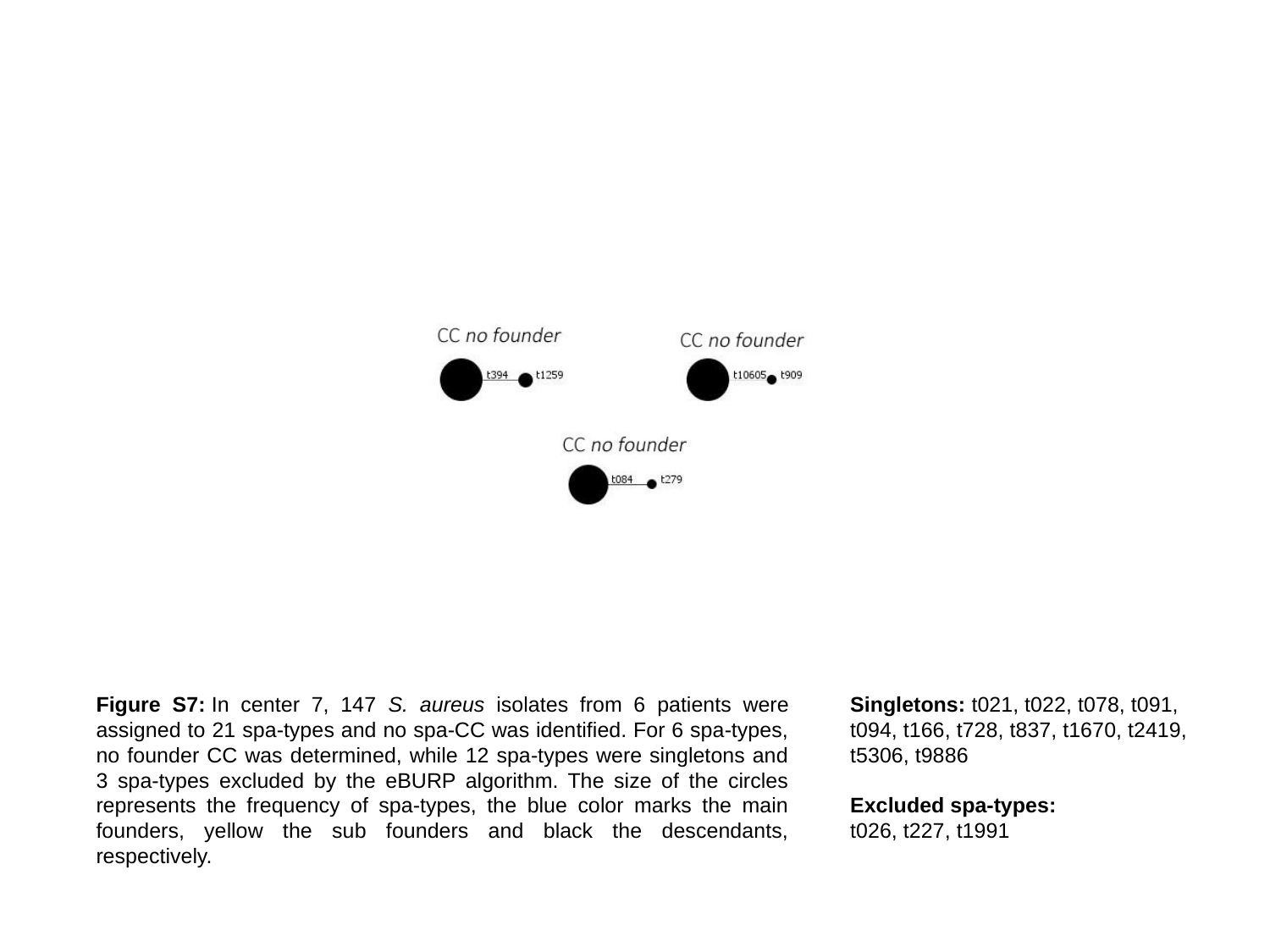

Singletons: t021, t022, t078, t091,
t094, t166, t728, t837, t1670, t2419,
t5306, t9886
Excluded spa-types:
t026, t227, t1991
Figure S7: In center 7, 147 S. aureus isolates from 6 patients were assigned to 21 spa-types and no spa-CC was identified. For 6 spa-types, no founder CC was determined, while 12 spa-types were singletons and 3 spa-types excluded by the eBURP algorithm. The size of the circles represents the frequency of spa-types, the blue color marks the main founders, yellow the sub founders and black the descendants, respectively.

## Slide 8
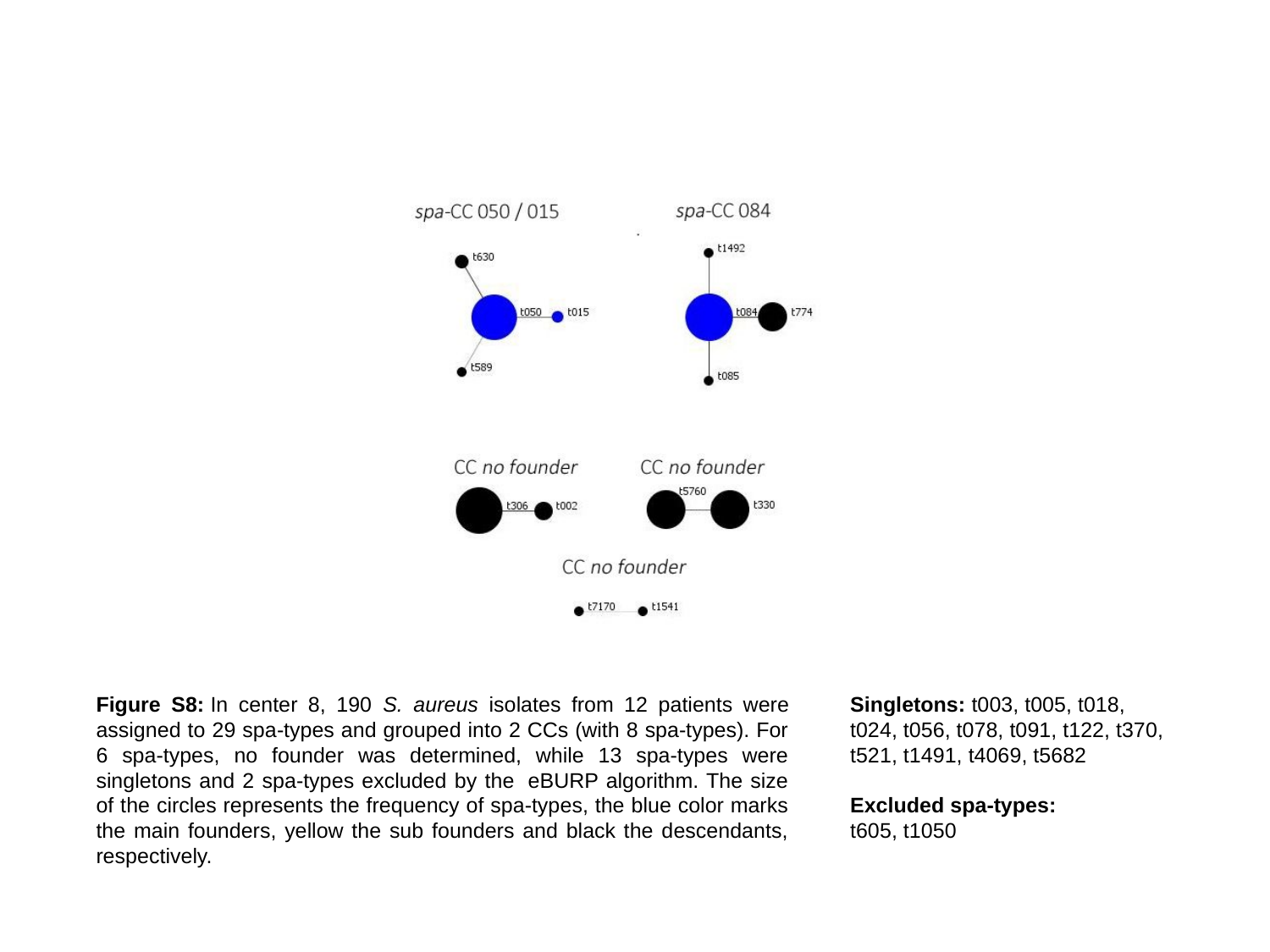

Singletons: t003, t005, t018,
t024, t056, t078, t091, t122, t370,
t521, t1491, t4069, t5682
Excluded spa-types:
t605, t1050
Figure S8: In center 8, 190 S. aureus isolates from 12 patients were assigned to 29 spa-types and grouped into 2 CCs (with 8 spa-types). For 6 spa-types, no founder was determined, while 13 spa-types were singletons and 2 spa-types excluded by the  eBURP algorithm. The size of the circles represents the frequency of spa-types, the blue color marks the main founders, yellow the sub founders and black the descendants, respectively.

## Slide 9
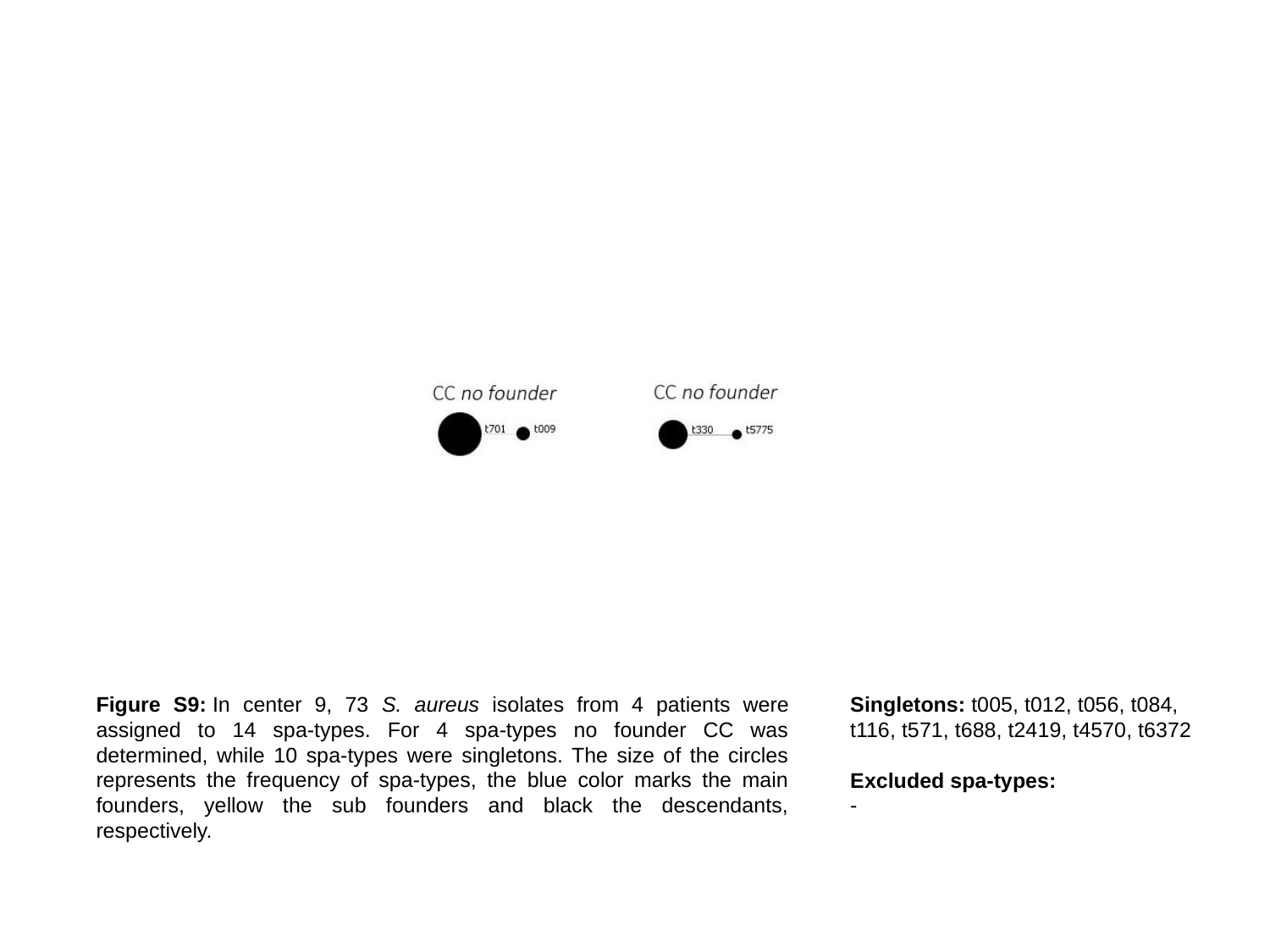

Singletons: t005, t012, t056, t084,
t116, t571, t688, t2419, t4570, t6372
Excluded spa-types:
-
Figure S9: In center 9, 73 S. aureus isolates from 4 patients were assigned to 14 spa-types. For 4 spa-types no founder CC was determined, while 10 spa-types were singletons. The size of the circles represents the frequency of spa-types, the blue color marks the main founders, yellow the sub founders and black the descendants, respectively.

## Slide 10
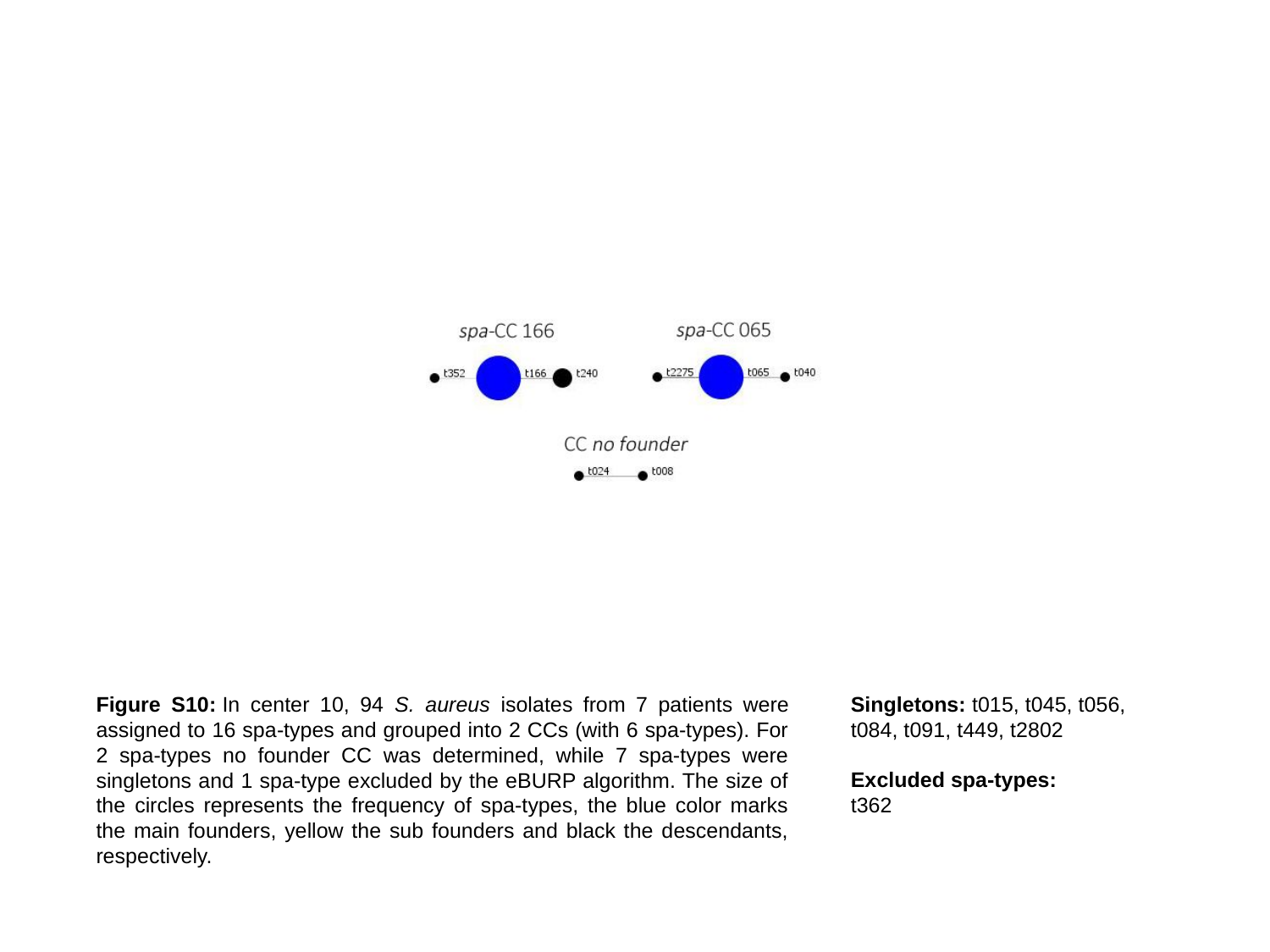

Singletons: t015, t045, t056,
t084, t091, t449, t2802
Excluded spa-types:
t362
Figure S10: In center 10, 94 S. aureus isolates from 7 patients were assigned to 16 spa-types and grouped into 2 CCs (with 6 spa-types). For 2 spa-types no founder CC was determined, while 7 spa-types were singletons and 1 spa-type excluded by the eBURP algorithm. The size of the circles represents the frequency of spa-types, the blue color marks the main founders, yellow the sub founders and black the descendants, respectively.

## Slide 11
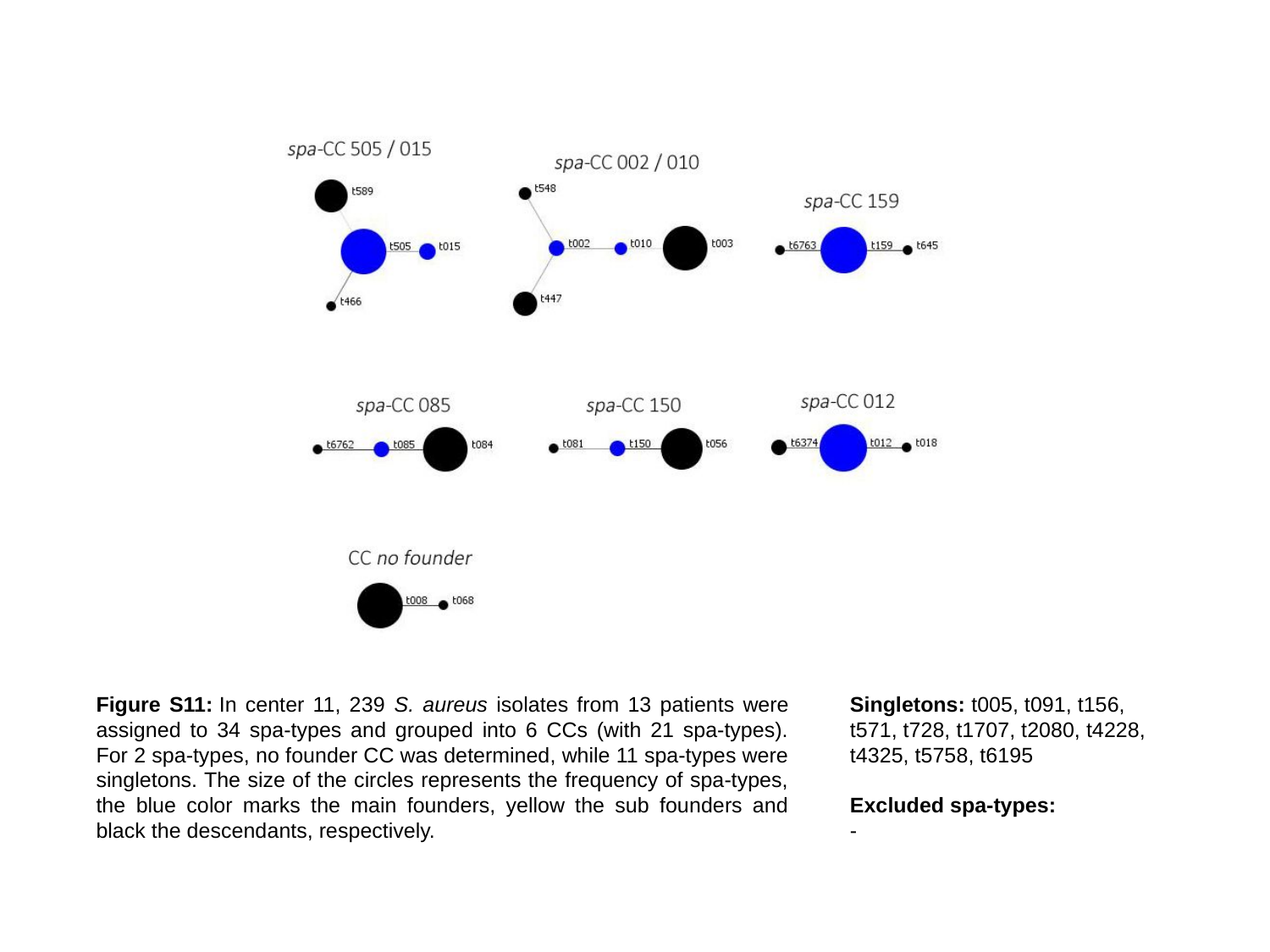

Singletons: t005, t091, t156,
t571, t728, t1707, t2080, t4228,
t4325, t5758, t6195
Excluded spa-types:
-
Figure S11: In center 11, 239 S. aureus isolates from 13 patients were assigned to 34 spa-types and grouped into 6 CCs (with 21 spa-types). For 2 spa-types, no founder CC was determined, while 11 spa-types were singletons. The size of the circles represents the frequency of spa-types, the blue color marks the main founders, yellow the sub founders and black the descendants, respectively.

## Slide 12
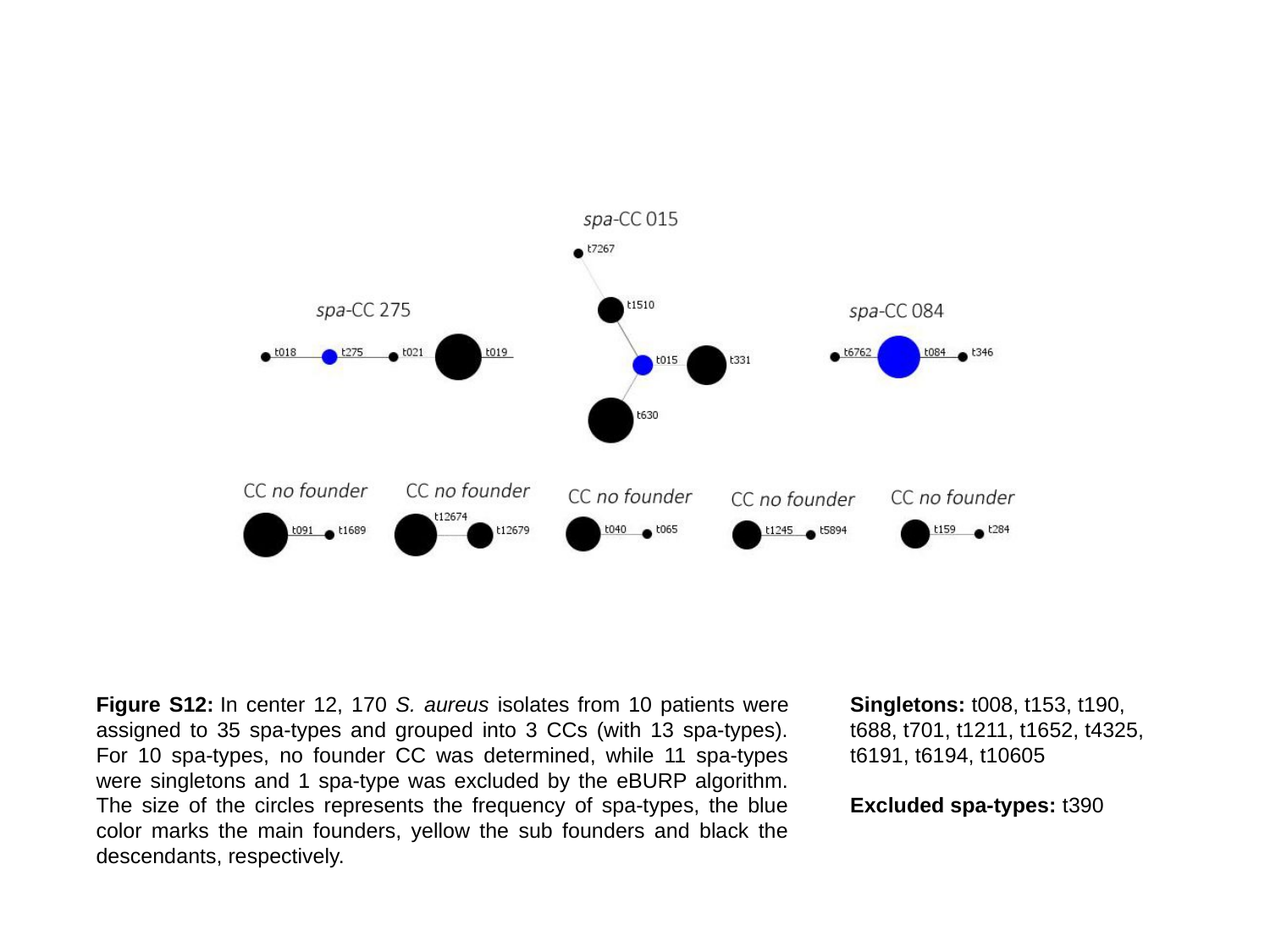

Singletons: t008, t153, t190,
t688, t701, t1211, t1652, t4325,
t6191, t6194, t10605
Excluded spa-types: t390
Figure S12: In center 12, 170 S. aureus isolates from 10 patients were assigned to 35 spa-types and grouped into 3 CCs (with 13 spa-types). For 10 spa-types, no founder CC was determined, while 11 spa-types were singletons and 1 spa-type was excluded by the eBURP algorithm. The size of the circles represents the frequency of spa-types, the blue color marks the main founders, yellow the sub founders and black the descendants, respectively.

## Slide 13
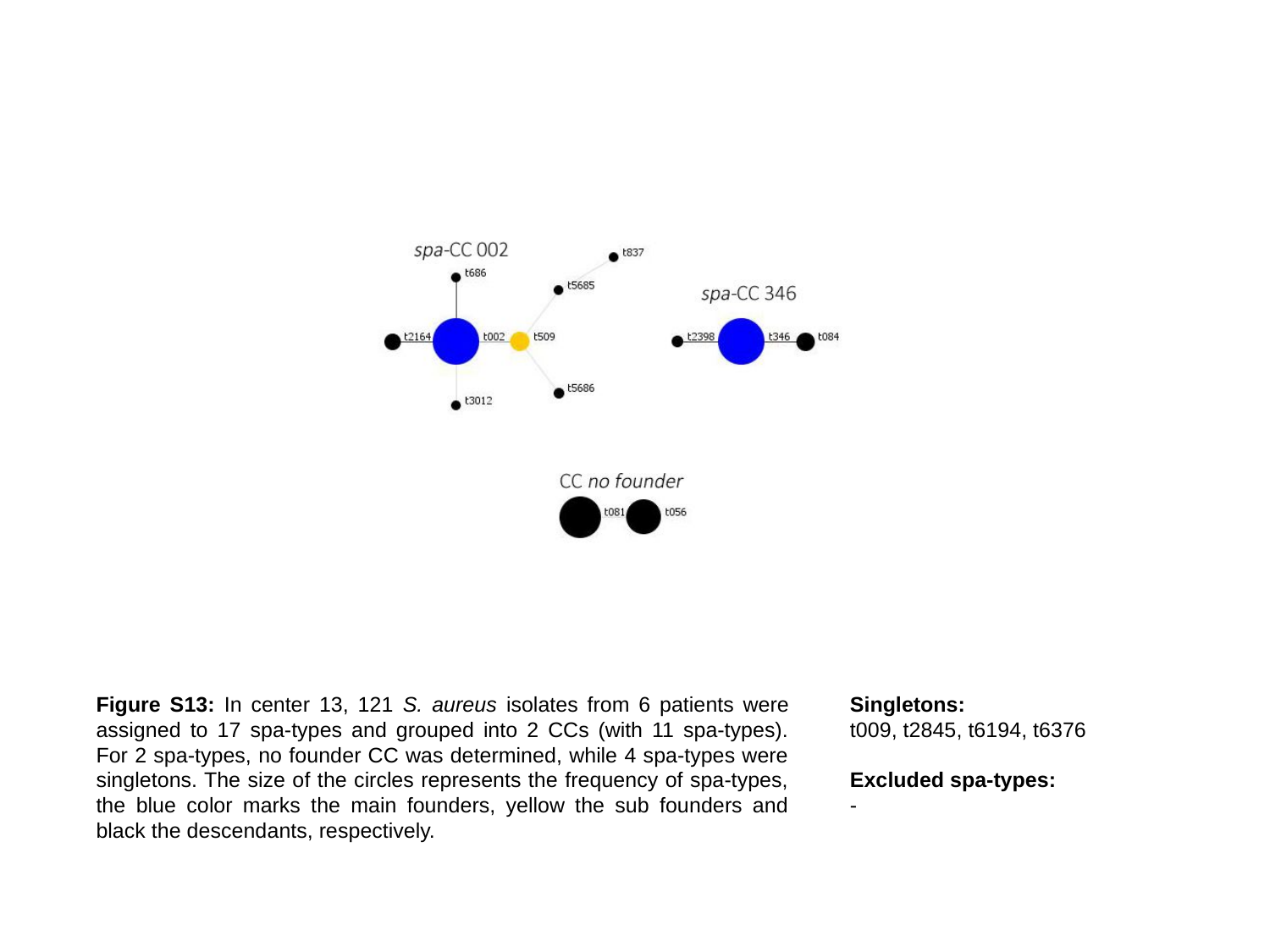

Singletons:
t009, t2845, t6194, t6376
Excluded spa-types:
-
Figure S13: In center 13, 121 S. aureus isolates from 6 patients were assigned to 17 spa-types and grouped into 2 CCs (with 11 spa-types). For 2 spa-types, no founder CC was determined, while 4 spa-types were singletons. The size of the circles represents the frequency of spa-types, the blue color marks the main founders, yellow the sub founders and black the descendants, respectively.

## Slide 14
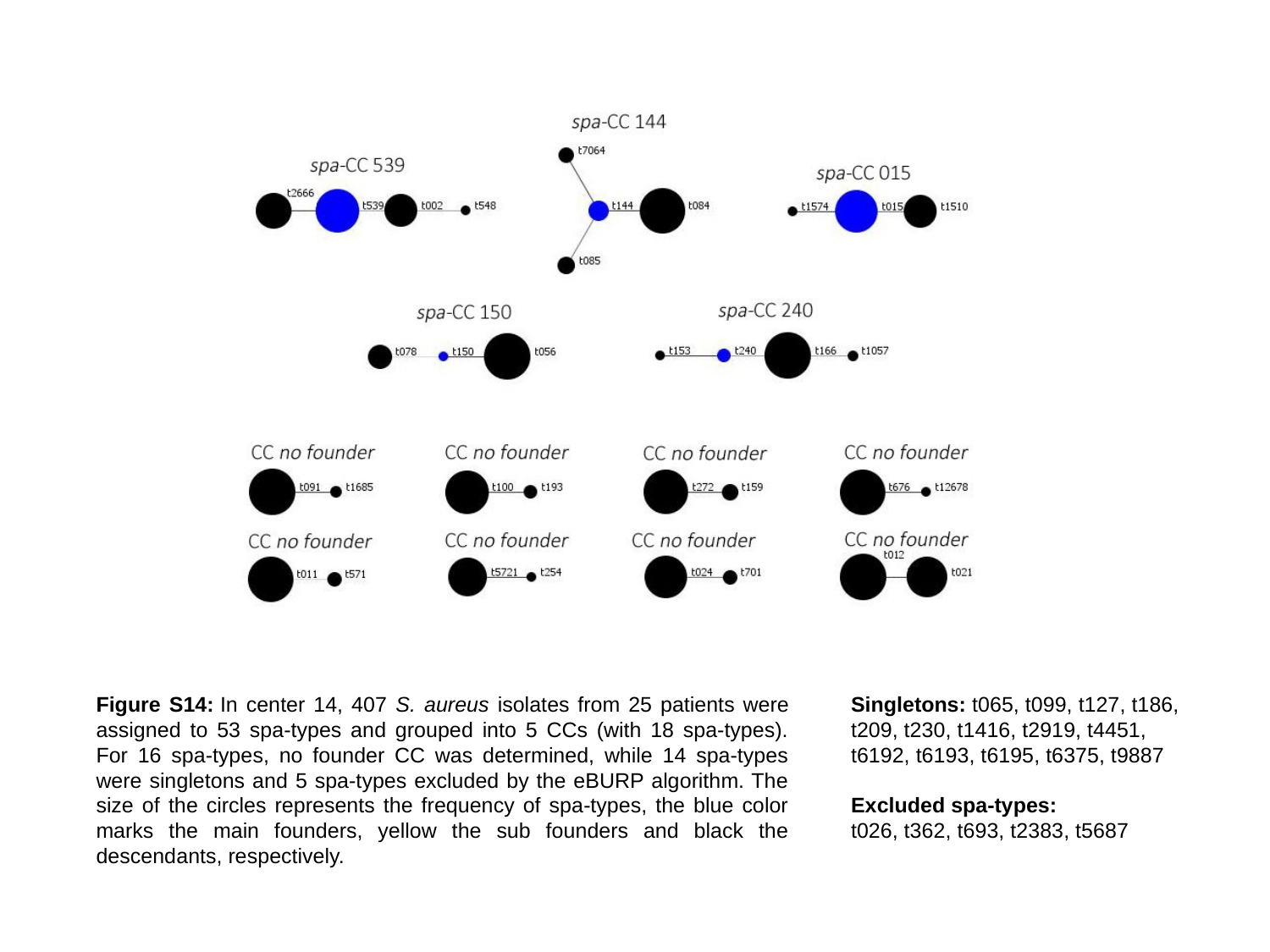

Singletons: t065, t099, t127, t186,
t209, t230, t1416, t2919, t4451,
t6192, t6193, t6195, t6375, t9887
Excluded spa-types:
t026, t362, t693, t2383, t5687
Figure S14: In center 14, 407 S. aureus isolates from 25 patients were assigned to 53 spa-types and grouped into 5 CCs (with 18 spa-types). For 16 spa-types, no founder CC was determined, while 14 spa-types were singletons and 5 spa-types excluded by the eBURP algorithm. The size of the circles represents the frequency of spa-types, the blue color marks the main founders, yellow the sub founders and black the descendants, respectively.

## Slide 15
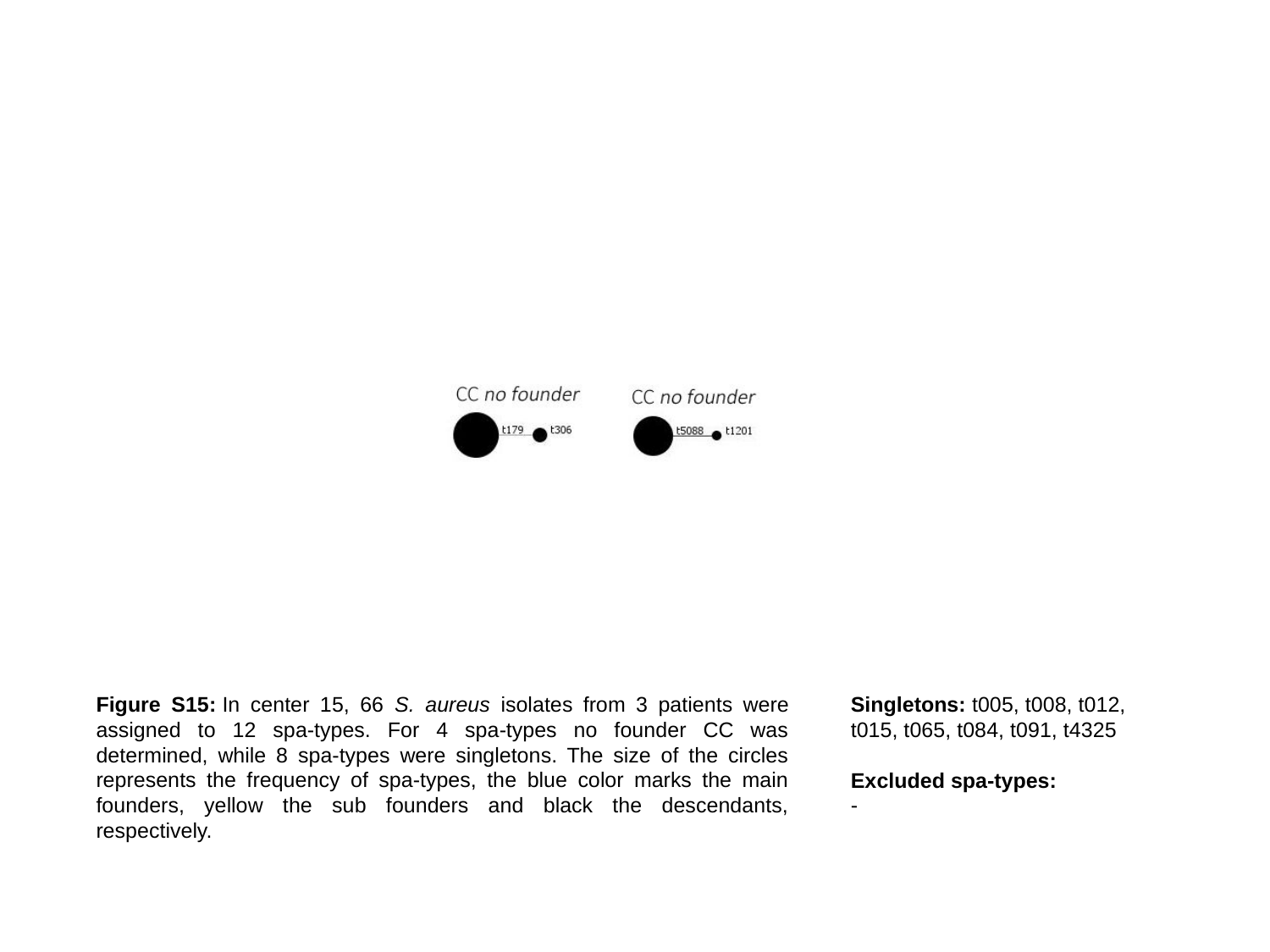

Singletons: t005, t008, t012,
t015, t065, t084, t091, t4325
Excluded spa-types:
-
Figure S15: In center 15, 66 S. aureus isolates from 3 patients were assigned to 12 spa-types. For 4 spa-types no founder CC was determined, while 8 spa-types were singletons. The size of the circles represents the frequency of spa-types, the blue color marks the main founders, yellow the sub founders and black the descendants, respectively.

## Slide 16
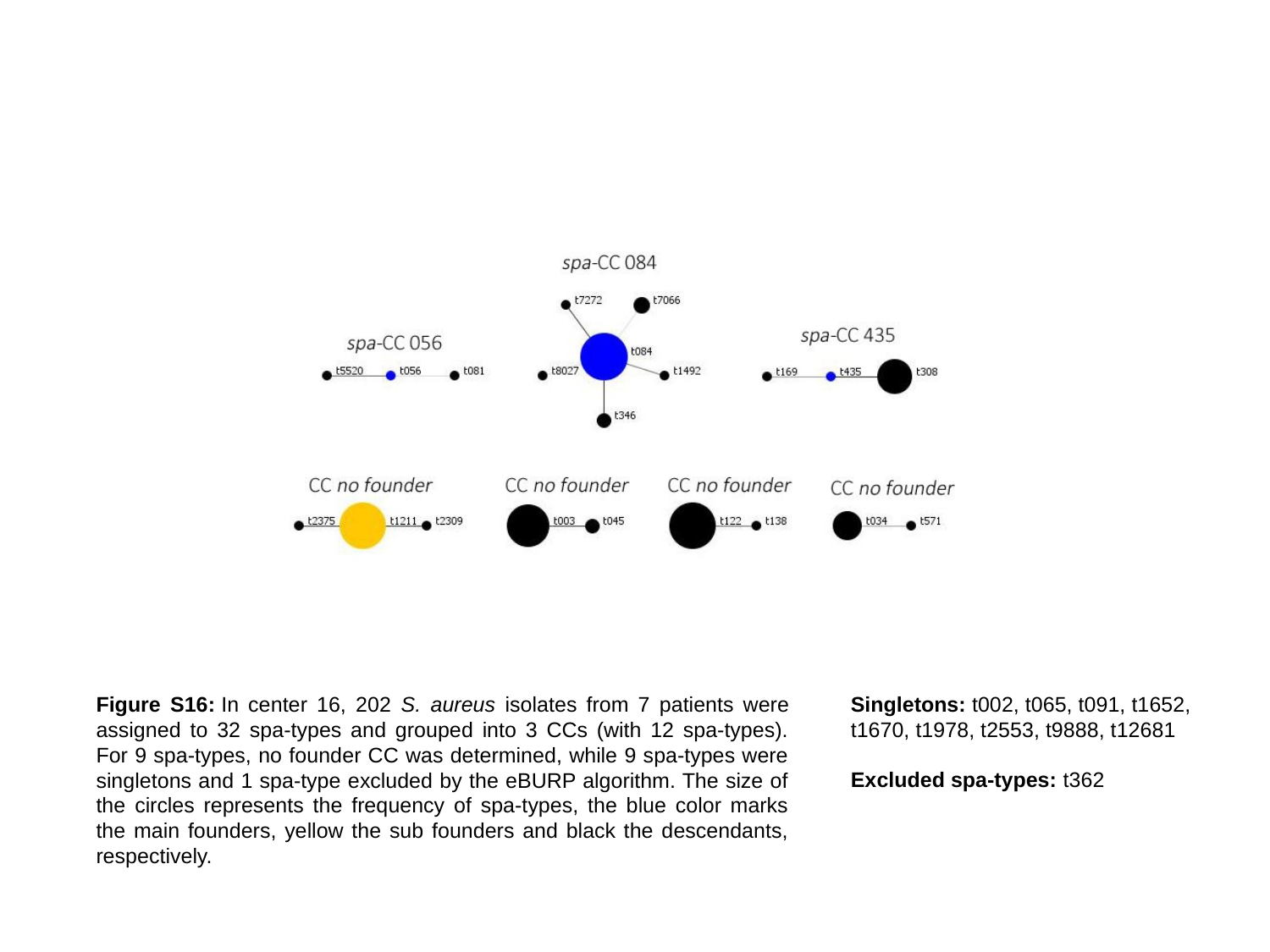

Singletons: t002, t065, t091, t1652,
t1670, t1978, t2553, t9888, t12681
Excluded spa-types: t362
Figure S16: In center 16, 202 S. aureus isolates from 7 patients were assigned to 32 spa-types and grouped into 3 CCs (with 12 spa-types). For 9 spa-types, no founder CC was determined, while 9 spa-types were singletons and 1 spa-type excluded by the eBURP algorithm. The size of the circles represents the frequency of spa-types, the blue color marks the main founders, yellow the sub founders and black the descendants, respectively.

## Slide 17
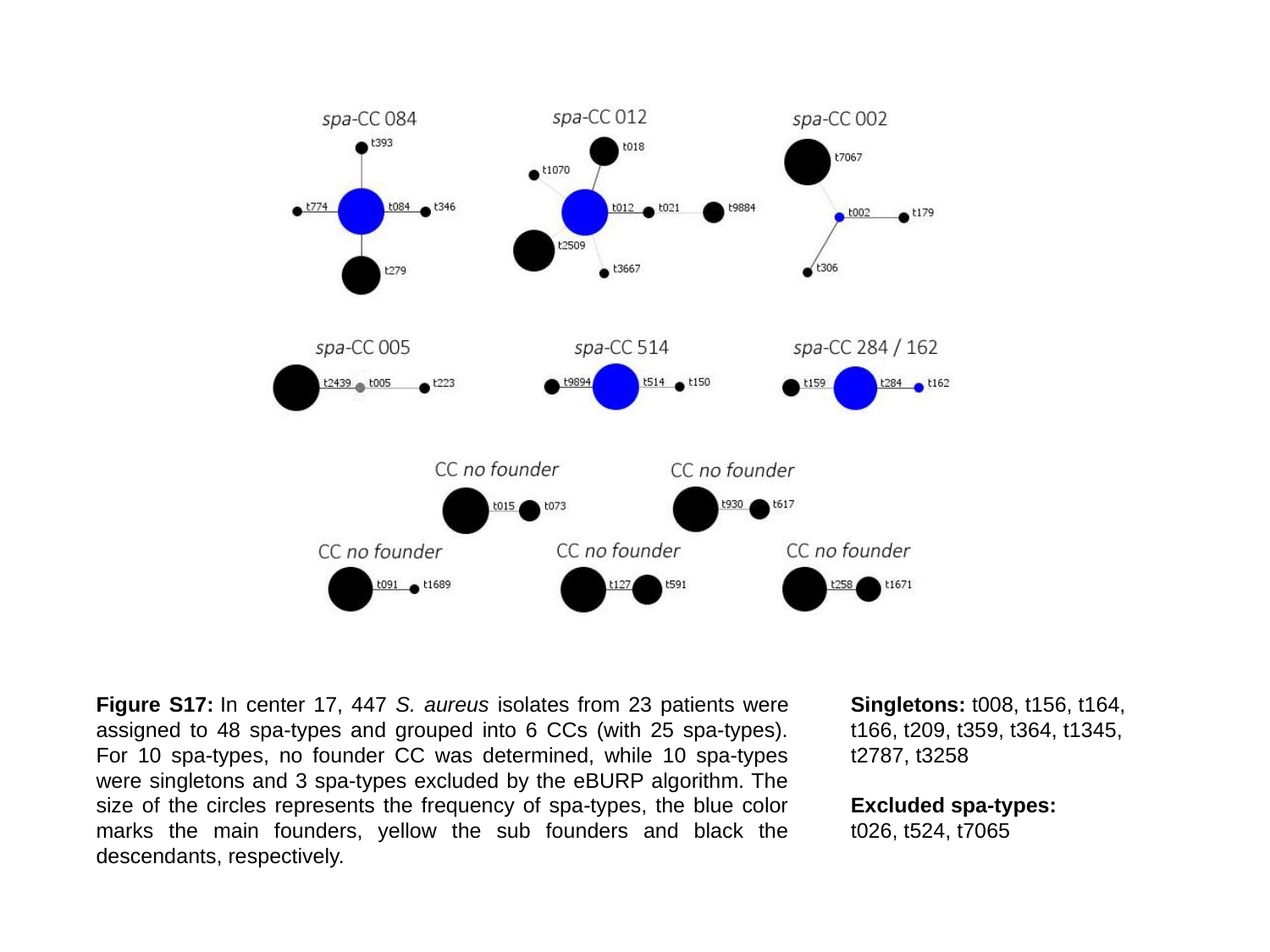

Singletons: t008, t156, t164,
t166, t209, t359, t364, t1345,
t2787, t3258
Excluded spa-types:
t026, t524, t7065
Figure S17: In center 17, 447 S. aureus isolates from 23 patients were assigned to 48 spa-types and grouped into 6 CCs (with 25 spa-types). For 10 spa-types, no founder CC was determined, while 10 spa-types were singletons and 3 spa-types excluded by the eBURP algorithm. The size of the circles represents the frequency of spa-types, the blue color marks the main founders, yellow the sub founders and black the descendants, respectively.
